# Supplementary material for: Repeat‐associated non‐AUG translation in C9orf72‐ALS/FTD is driven by neuronal excitation and stress
Source: EMBO Mol Med. 2019 Jan 7;11(2):e9423. doi: 10.15252/emmm.201809423 (PMC6365928; doi:10.15252/emmm.201809423)
Supplement: Supplementary file 1 — Appendix [file EMMM-11-e9423-s001.pdf]

Table of Contents – Appendix Table S1

Figure 1 — *2*
- 1B DPR level (relative to GA) — 2
- 1D DPR positive cells (%) — 2
- 1D Fluorescent Intensity — 2

Figure EV1 — *3*
- 1B DPR level (relative to GA) — 3
- 1D DPR positive cells (%) — 3
- 1D Fluorescent Intensity — 4

Figure 2 — *5*
- 2A (SSP dose response curve) — 5
- 2B Western Blot DPR level — 5
- 2C NSC34 Fluorescent Intensity — 5
- 2C Cortical Neuron Fluorescent Intensity — 6
- 2D iMN Fluorescent Intensity — 6

Figure EV2 — *7*

Figure 3 — *7*
- 3B Cortical Neuron Fluorescent Intensity — 7
- 3B iMN Fluorescent Intensity — 8

Figure 4 — *8*
- 4C Fluorescent Intensity — 8

Figure 5/Figure EV5 — *8*
- Figure 5A and Figure EV5B p-eif2α/eif2α protein levels — 8
- Figure 5A and Figure EV5B PERK protein levels — 9
- Figure 5A and Figure EV5B ATF4 protein levels — 9

Figure 6 — *9*
- Figure 6A NSC34 RAN Inhibitor Fluorescent Intensity — 9
- Figure 6B Cortical Neuron RAN Inhibitor Fluorescent Intensity — 10

**Figure 1**				
**1B DPR level (relative to GA)**				
Uncorrected Fisher's LSD	Mean Diff.	Significant?	Summary	Individual P Value
GA vs. GP	0.2732	No	ns	0.1258
GA vs. GR	0.6552	Yes	***	0.0009
**1D DPR positive cells (%)**				
A10				
Dunnett's multiple comparison	Mean Diff.	95% CI of diff.	Significant?	Summary
GA				
HEK vs. NSC34	21.35	6.186 to 36.51	Yes	**
HEK vs. Neurons	25.43	10.27 to 40.59	Yes	***
GP				
HEK vs. NSC34	25.44	10.28 to 40.60	Yes	***
HEK vs. Neurons	29.67	14.51 to 44.83	Yes	****
GR				
HEK vs. NSC34	26.38	11.22 to 41.54	Yes	***
HEK vs. Neurons	29.27	14.11 to 44.43	Yes	****
**1D Fluorescent Intensity**				
Uncorrected Fisher's LSD	Mean Diff.	Significant?	Summary	Individual P Value
HEK:GA vs. HEK:GP	0.251	Yes	*	0.0281
HEK:GA vs. HEK:GR	0.512	Yes	****	< 0.0001
HEK:GA vs. NSC34:GA	0.615	Yes	****	< 0.0001
HEK:GA vs. NSC34:GP	0.749	Yes	****	< 0.0001
HEK:GA vs. NSC34:GR	0.837	Yes	****	< 0.0001
HEK:GA vs. Neurons:GA	0.721	Yes	****	< 0.0001
HEK:GA vs. Neurons:GP	0.812	Yes	****	< 0.0001
HEK:GA vs. Neurons:GR	0.878	Yes	****	< 0.0001
HEK:GP vs. HEK:GR	0.261	Yes	*	0.0225
HEK:GP vs. NSC34:GA	0.364	Yes	**	0.0017
HEK:GP vs. NSC34:GP	0.498	Yes	****	< 0.0001
HEK:GP vs. NSC34:GR	0.586	Yes	****	< 0.0001
HEK:GP vs. Neurons:GA	0.47	Yes	****	< 0.0001

HEK:GP vs. Neurons:GP	0.561	Yes	****	< 0.0001
HEK:GP vs. Neurons:GR	0.627	Yes	****	< 0.0001
HEK:GR vs. NSC34:GA	0.103	No	ns	0.3615
HEK:GR vs. NSC34:GP	0.237	Yes	*	0.0378
HEK:GR vs. NSC34:GR	0.325	Yes	**	0.0049
HEK:GR vs. Neurons:GA	0.209	No	ns	0.0662
HEK:GR vs. Neurons:GP	0.3	Yes	**	0.0091
HEK:GR vs. Neurons:GR	0.366	Yes	**	0.0016
NSC34:GA vs. NSC34:GP	0.134	No	ns	0.236
NSC34:GA vs. NSC34:GR	0.222	No	ns	0.0513
NSC34:GA vs. Neurons:GA	0.106	No	ns	0.3478
NSC34:GA vs. Neurons:GP	0.197	No	ns	0.083
NSC34:GA vs. Neurons:GR	0.263	Yes	*	0.0216
NSC34:GP vs. NSC34:GR	0.088	No	ns	0.4353
NSC34:GP vs. Neurons:GA	-0.028	No	ns	0.8036
NSC34:GP vs. Neurons:GP	0.063	No	ns	0.5761
NSC34:GP vs. Neurons:GR	0.129	No	ns	0.2538
NSC34:GR vs. Neurons:GA	-0.116	No	ns	0.3044
NSC34:GR vs. Neurons:GP	-0.025	No	ns	0.8243
NSC34:GR vs. Neurons:GR	0.041	No	ns	0.7158
Neurons:GA vs. Neurons:GP	0.091	No	ns	0.4199
Neurons:GA vs. Neurons:GR	0.157	No	ns	0.1657
Neurons:GP vs. Neurons:GR	0.066	No	ns	0.5581

**Figure EV1**					
**1B DPR level (relative to GA)**					
Uncorrected Fisher's LSD	Mean Diff.	Significant?	Summary	Individual P Value	
PA vs. PG	-0.76	No	ns	0.1268	
PA vs. PR	-0.27	No	ns	0.5708	
**1D DPR positive cells (%)**					
Dunnett's multiple comparisons tes	Mean Diff.	95% CI of diff.	Significan	Summary	
PA					
HEK vs. NSC34	28.87	12.00 to 45.74	Yes	***	
HEK vs. Neurons	32.72	15.85 to 49.59	Yes	****	
PG					
HEK vs. NSC34	35.61	18.74 to 52.48	Yes	****	

HEK vs. Neurons	39.22	22.35 to 56.09	Yes	****	
PR					
HEK vs. NSC34	29.22	12.35 to 46.09	Yes	***	
HEK vs. Neurons	32.17	15.30 to 49.04	Yes	****	
**1D Fluorescent Intensity**					
Uncorrected Fisher's LSD	Mean Diff.	Significant?	Summary	Individual P Value	
HEK:PA vs. HEK:PG	-0.272	No	ns	0.2723	
HEK:PA vs. HEK:PR	-0.01	No	ns	0.9677	
HEK:PA vs. NSC34:PA	0.601	Yes	*	0.0168	
HEK:PA vs. NSC34:PG	0.534	Yes	*	0.0329	
HEK:PA vs. NSC34:PR	0.636	Yes	*	0.0116	
HEK:PA vs. Neurons:PA	0.796	Yes	**	0.0018	
HEK:PA vs. Neurons:PG	0.661	Yes	**	0.0088	
HEK:PA vs. Neurons:PR	0.723	Yes	**	0.0043	
HEK:PG vs. HEK:PR<br>A37	0.262	No	ns	0.2902	
HEK:PG vs. NSC34:PA	0.873	Yes	***	0.0007	
HEK:PG vs. NSC34:PG	0.806	Yes	**	0.0016	
HEK:PG vs. NSC34:PR	0.908	Yes	***	0.0004	
HEK:PG vs. Neurons:PA	1.068	Yes	****	< 0.0001	
HEK:PG vs. Neurons:PG	0.933	Yes	***	0.0003	
HEK:PG vs. Neurons:PR	0.995	Yes	***	0.0001	
HEK:PR vs. NSC34:PA	0.611	Yes	*	0.0151	
HEK:PR vs. NSC34:PG	0.544	Yes	*	0.0299	
HEK:PR vs. NSC34:PR	0.646	Yes	*	0.0104	
HEK:PR vs. Neurons:PA	0.806	Yes	**	0.0016	
HEK:PR vs. Neurons:PG	0.671	Yes	**	0.0078	
HEK:PR vs. Neurons:PR	0.733	Yes	**	0.0038	
NSC34:PA vs. NSC34:PG	-0.067	No	ns	0.7861	
NSC34:PA vs. NSC34:PR	0.035	No	ns	0.8873	
NSC34:PA vs. Neurons:PA	0.195	No	ns	0.4305	
NSC34:PA vs. Neurons:PG	0.06	No	ns	0.808	
NSC34:PA vs. Neurons:PR	0.122	No	ns	0.6214	
NSC34:PG vs. NSC34:PR	0.102	No	ns	0.6796	
NSC34:PG vs. Neurons:PA	0.262	No	ns	0.2902	
NSC34:PG vs. Neurons:PG	0.127	No	ns	0.6072	
NSC34:PG vs. Neurons:PR	0.189	No	ns	0.4447	

NSC34:PR vs. Neurons:PA	0.16	No	ns	0.5174	
NSC34:PR vs. Neurons:PG	0.025	No	ns	0.9193	
NSC34:PR vs. Neurons:PR	0.087	No	ns	0.7246	
Neurons:PA vs. Neurons:PG	-0.135	No	ns	0.5848	
Neurons:PA vs. Neurons:PR	-0.073	No	ns	0.7675	
Neurons:PG vs. Neurons:PR	0.062	No	ns	0.8017	

**Figure 2**					
**2A (SSP dose response curve)**					
P value					
0.489796					
0.397368					
0.120726					
0.0100101					
0.0027665					
0.00510791					
**2B Western Blot DPR level**					
Uncorrected Fisher's L	Mean Diff.	Significant?	Summary	Individual P Value	
CTRL vs. TG	-0.9653	Yes	**	0.0045	
CTRL vs. Mena	-0.9427	Yes	**	0.0055	
CTRL vs. SSP	-0.6797	Yes	*	0.0436	
CTRL vs. Diamide	-1.424	Yes	****	< 0.0001	
CTRL vs. CytoD	-0.8643	Yes	*	0.0107	
CTRL vs. MS-275	-1.135	Yes	***	0.0009	
CTRL vs. Etoposide	-1.364	Yes	****	< 0.0001	
CTRL vs. LeptoB	-0.7387	Yes	*	0.0285	
CTRL vs. HC	-1.258	Yes	***	0.0003	
CTRL vs. NaArs	-1.025	Yes	**	0.0026	
CTRL vs. TM	-0.6603	Yes	*	0.0498	
CTRL vs. Glut	-0.402	No	ns	0.2299	
**2C NSC34 Fluorescent Intensity**					
Uncorrected Fisher's L	Mean Diff.	Significant?	Summary	Individual P Value	
CTRL vs. TG	-0.5336	No	ns	0.0722	
CTRL vs. Mena	-0.926	Yes	**	0.0019	
CTRL vs. SSP	-0.8743	Yes	**	0.0033	
CTRL vs. Diamide	-0.9583	Yes	**	0.0013	F35

CTRL vs. CytoD	-0.1551	No	ns	0.6006	
CTRL vs. MS 275	-1.196	Yes	****	< 0.0001	
CTRL vs. Etoposide	-1.288	Yes	****	< 0.0001	
CTRL vs. Lepto B	-1.071	Yes	***	0.0003	
CTRL vs. HC	-0.791	Yes	**	0.0036	
CTRL vs. NaArs	-1.37	Yes	****	< 0.0001	
CTRL vs. TM	-1.022	Yes	***	0.0006	
CTRL vs. Glut	-0.85	Yes	**	0.0018	

**2C Cortical Neuron Fluorescent Intensity**						
Uncorrected Fisher's L	Mean Diff.	95% CI of diff.	Significan	Summary	Individual P Value	
CTRL vs. TG	-0.455	-0.9506 to 0.0406	No	ns	0.0718	
CTRL vs. Mena	-0.811	-1.307 to -0.3154	Yes	**	0.0014	
CTRL vs. SSP	-0.6807	-1.176 to -0.1850	Yes	**	0.0073	
CTRL vs. Diamide	-0.8013	-1.297 to -0.3057	Yes	**	0.0016	
CTRL vs. CytoD	-0.3313	-0.8270 to 0.1643	No	ns	0.1893	
CTRL vs. MS 275	-0.8907	-1.386 to -0.3950	Yes	***	0.0005	
CTRL vs. Etoposide	-0.9083	-1.404 to -0.4127	Yes	***	0.0004	
CTRL vs. Lepto B	-0.8963	-1.392 to -0.4007	Yes	***	0.0004	
CTRL vs. HC	-1.237	-1.733 to -0.7417	Yes	****	< 0.0001	
CTRL vs. NaArs	-0.6433	-1.139 to -0.1477	Yes	*	0.0112	
CTRL vs. TM	-0.674	-1.170 to -0.1784	Yes	**	0.0079	
CTRL vs. Glut	-1.671	-2.166 to -1.175	Yes	****	< 0.0001	

**2D iMN Fluorescent Intensity**					
Uncorrected Fisher's L	Mean Diff.	Significant?	Summary	Individual P Value	
CTRL vs. TG	-0.6899	Yes	*	0.0499	
CTRL vs. Mena	-0.7481	Yes	*	0.0336	
CTRL vs. SSP	-0.9522	Yes	**	0.0071	
CTRL vs. Diamide	-1.231	Yes	***	0.0006	
CTRL vs. CytoD	-0.1805	No	ns	0.6059	
CTRL vs. MS 275	-1.075	Yes	**	0.0024	
CTRL vs. Etoposide	-1.212	Yes	***	0.0007	
CTRL vs. Lepto B	-1.352	Yes	***	0.0002	
CTRL vs. HC	-1.1	Yes	**	0.002	
CTRL vs. NaArs	-0.9612	Yes	**	0.0066	
CTRL vs. TM	-1.103	Yes	**	0.0019	

CTRL vs. Glut	-1.591	Yes	****	< 0.0001		

**Figure EV2**							
Dose Response P Values							
Thapsigargin	Tunicamycin	Allyl Alcohol	Menadione	Bromobenzene	Staurosporine	Cytochalasin D	Diamide
P value	P value	P value	P value	P value	P value	P value	P value
0.79850	0.89501	0.83076	0.02588	0.60410	0.48980	0.69757	0.96366
0.89975	0.91302	0.05530	0.02761	0.15765	0.39737	0.97776	0.44257
0.27209	0.65106	0.00512	0.00112	0.02448	0.12073	0.99662	0.08587
0.00817	0.11078	0.00200	0.00024	0.00107	0.01001	0.93672	0.05030
0.00940	0.00663	0.00073	0.00096	0.00890	0.00277	0.99541	0.07009
0.00562	0.02762	0.00193	< 0.0001	0.00166	0.00511	0.66968	0.01286
MS-275	Etoposide	Leptomycin B	Homocysteine	Paraquat	Sodium Arsenite	H2O2	KCl
P value	P value	P value	P value	P value	P value	P value	P value
0.56877	0.92861	0.35717	0.01878	0.71362	0.66536	0.39229	0.39085
0.33083	0.67932	0.03656	0.01497	0.38362	0.13143	0.10127	0.45610
0.11976	0.19514	0.00681	0.00321	0.04408	0.00236	0.03630	0.03093
0.16729	0.03325	0.00514	0.00175	0.04690	0.00480	0.05867	0.07132
0.01891	0.07951	0.00084	0.00780	0.00670	0.03177	0.00516	0.04922
0.04607	0.04343	0.00490	0.00096	0.01236	0.00258	0.03712	0.04145
Glutamate							
P value							
0.54452							
0.04506							
0.00512							
0.08640							
0.02440							
0.00589							

**Figure 3**				
**3B Cortical Neuron Fluorescent Intensity**				
Uncorrected Fisher's LSD	Mean Diff.	Significant?	Summary	Individual P Value
CTRL vs. Glut	-1.675	Yes	****	< 0.0001
CTRL vs. Glut + MK801	-0.3317	No	ns	0.1637
CTRL vs. Glut + NBQX	-0.756	Yes	**	0.0016
CTRL vs. Glut + Both	-0.3137	No	ns	0.1877
CTRL vs. AMPA	-0.7414	Yes	**	0.002
CTRL vs. AMPA + NBQX	-0.4517	No	ns	0.0583
CTRL vs. NMDA	-0.9967	Yes	****	< 0.0001
CTRL vs. NMDA + MK801	-0.165	No	ns	0.4876
CTRL vs. HC	-1.241	Yes	****	< 0.0001

CTRL vs. HC + both	-0.454	No	ns	0.057
**3B iMN Fluorescent Intensity**				
Uncorrected Fisher's LSD	Mean Diff.	Significant?	Summary	Individual P Value
CTRL vs. Glut	-1.591	Yes	****	< 0.0001
CTRL vs. Glut + MK801	-0.4672	No	ns	0.1787
CTRL vs. Glut + NBQX	-1.002	Yes	**	0.0044
CTRL vs. Glut + Both	-0.5446	No	ns	0.1175
CTRL vs. AMPA	-0.7702	Yes	*	0.0275
CTRL vs. AMPA + NBQX	-0.6216	No	ns	0.0744
CTRL vs. NMDA	-1.352	Yes	***	0.0001
CTRL vs. NMDA + MK801	-0.5842	No	ns	0.0933
CTRL vs. HC	-1.1	Yes	**	0.0018
CTRL vs. HC + both	-0.3176	No	ns	0.3598

**Figure 4**				
**4C Fluorescent Intensity**				
Dunnett's multiple comparisons test	Mean Diff.	95% CI of diff.	Significant?	Summary
No Stim vs. Low Stim	-0.005654	-0.2833 to 0.2720	No	ns
No Stim vs. Med Stim	-0.6513	-0.9226 to -0.3800	Yes	****
No Stim vs. High Stim	-0.5418	-0.8094 to -0.2741	Yes	****

**Figure 5/Figure EV5**				
**Figure 5A and Figure EV5B p-eif2α/eif2α protein levels**				
Uncorrected Fisher's LSD	Mean Diff.	Significant?	Summary	Individual P Value
CTRL vs. TG	-0.6357	Yes	*	0.0384
CTRL vs. Mena	-1.323	Yes	***	0.0001
CTRL vs. SSP	-0.9846	Yes	**	0.0023
CTRL vs. Diamide	-0.9271	Yes	**	0.0038
CTRL vs. CytoD	-0.1262	No	ns	0.6686
CTRL vs. MS 275	-0.8677	Yes	**	0.0062
CTRL vs. Etoposide	-1.102	Yes	***	0.0008
CTRL vs. Lepto B	-1.229	Yes	***	0.0003
CTRL vs. HC	-0.635	Yes	*	0.0386
CTRL vs. NaArs	-0.8602	Yes	**	0.0066
CTRL vs. TM	-0.735	Yes	*	0.0181
CTRL vs. Glut	-0.7169	Yes	*	0.0209

**Figure 5A and Figure EV5B PERK protein levels**				
Uncorrected Fisher's LSD	Mean Diff.	Significant?	Summary	Individual P Value
CTRL vs. TG	-1.458	Yes	**	0.0011
CTRL vs. Mena	-1.795	Yes	***	0.0001
CTRL vs. SSP	-0.8227	Yes	*	0.0485
CTRL vs. Diamide	-1.433	Yes	**	0.0013
CTRL vs. CytoD	-0.8531	Yes	*	0.0413
CTRL vs. MS 275	-1.231	Yes	**	0.0046
CTRL vs. Etoposide	-1.632	Yes	***	0.0004
CTRL vs. Lepto B	-1.439	Yes	**	0.0012
CTRL vs. HC	-1.59	Yes	***	0.0005
CTRL vs. NaArs	-1.41	Yes	**	0.0015
CTRL vs. TM	-1.649	Yes	***	0.0003
CTRL vs. Glut	-1.207	Yes	**	0.0054
**Figure 5A and Figure EV5B ATF4 protein levels**				
Uncorrected Fisher's LSD	Mean Diff.	Significant?	Summary	Individual P Value
CTRL vs. TG	-0.9352	Yes	***	0.0006
CTRL vs. Mena	-1.082	Yes	***	0.0001
CTRL vs. SSP	-0.52	Yes	*	0.0378
CTRL vs. Diamide	-0.551	Yes	*	0.0285
CTRL vs. CytoD	-0.495	Yes	*	0.0472
CTRL vs. MS 275	-1.532	Yes	****	< 0.0001
CTRL vs. Etoposide	-0.6106	Yes	*	0.0162
CTRL vs. Lepto B	-0.9276	Yes	***	0.0006
CTRL vs. HC	-1.085	Yes	***	0.0001
CTRL vs. NaArs	-1.189	Yes	****	< 0.0001
CTRL vs. TM	-0.6481	Yes	*	0.0113
CTRL vs. Glut	-0.5156	Yes	*	0.0393

**Figure 6**				
**Figure 6A NSC34 RAN Inhibitor Fluorescent Intensity**				
Uncorrected Fisher's LSD	Mean Diff.	Significant?	Summary	Individual P Value
Condition vs. +PERK	0.2211	Yes	***	0.0007
Condition vs. +CX5461	-0.09105	No	ns	0.1572
Condition vs. +Sal	-0.07155	No	ns	0.2657
Condition vs. +Cerca	0.1871	Yes	**	0.004
Condition vs. +Metf	-0.00205	No	ns	0.9745

Condition vs. +Traz	0.2383	Yes	***	0.0003
Condition vs. +1,3DBM	0.2794	Yes	****	< 0.0001
**Figure 6B Cortical Neuron RAN Inhibitor Fluorescent Intensity**				
Uncorrected Fisher's LSD	Mean Diff.	Significant?	Summary	Individual P Value
Glutamate vs. +PERKi	0.322	No	ns	0.0951
Glutamate vs. +CX5461	0.0499	No	ns	0.7689
Glutamate vs. +Sal	-0.1081	No	ns	0.5601
Glutamate vs. +Cerca	0.1317	No	ns	0.4831
Glutamate vs. +Metf	0.04175	No	ns	0.8292
Glutamate vs. +Traz	0.5144	Yes	**	0.005
Glutamate vs. +1,3DBM	0.5965	Yes	***	0.0005
