# Supplementary material for: Repeat‐associated non‐AUG translation in C9orf72‐ALS/FTD is driven by neuronal excitation and stress
Source: EMBO Mol Med. 2019 Jan 7;11(2):e9423. doi: 10.15252/emmm.201809423 (PMC6365928; doi:10.15252/emmm.201809423)
Supplement: Supplementary file 2 — Expanded View Figures PDF [file EMMM-11-e9423-s002.pdf]

Expanded View Figures

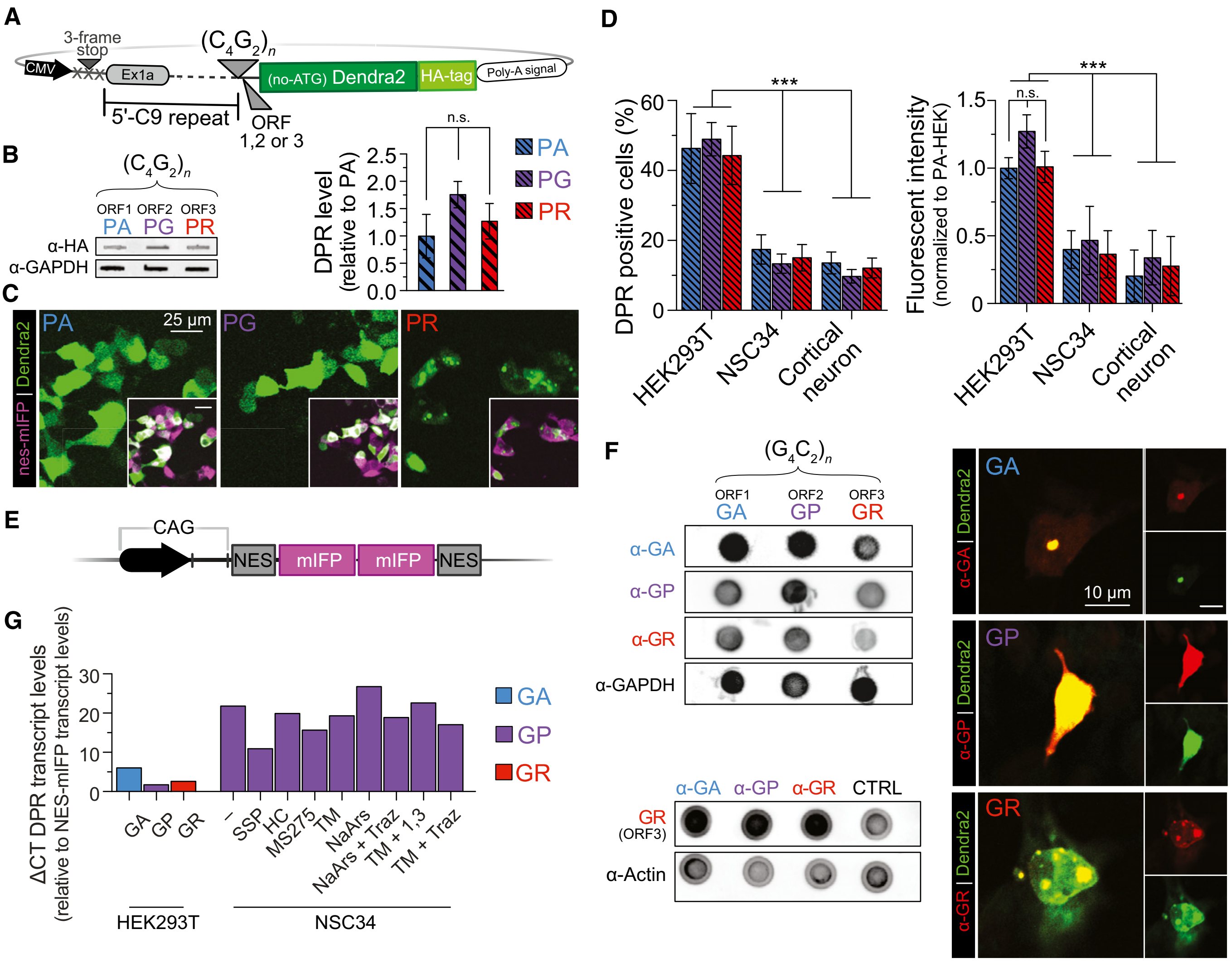

Figure EV1.

Figure EV1. *C9orf72*-based constructs containing the sense, $(C_4G_2)_{\sim 188}$, NRE undergo pathologically relevant non-AUG-dependent translation with DPR levels that vary by cell type.

A Plasmid reporter constructs developed to monitor non-AUG-dependent translation using a photoconvertible fluorescent protein, Dendra2. The endogenous *C9orf72* 5′ gene region of the sense direction is immediately upstream, and Dendra2 is immediately downstream of the NRE insertion site. In this construct, the sense sequence, $(G_4C_2)_{n\sim 188}$, was flipped to create the antisense sequence, $(C_4G_2)_{n\sim 188}$. Each construct is modified +1 or +2 base pairs to fluorescently monitor all individual ORFs that are in-frame with the Dendra2 and the HA affinity tag.

B All ORFs for the antisense coding, $(C_4G_2)_{n\sim 188}$, DPR, (PA), (PG), (PR), can be used to detect and quantify non-AUG-dependent translation using the c-terminal HA tag in a filter-trap binding assay. DPRs are detected in HEK293T cell 24 h post-transfection of the C9 DPR reporter construct. $n = 5$. Statistical comparisons were performed using a *t*-test with error bars representing the mean ± SEM.

C The *C9orf72* NRE non-AUG-dependent reporter constructs recapitulate localization patterns and pathological features identified in *C9orf72* NRE models and patients. The Dendra2 (green) c-terminal fusion was used to monitor cellular localization and fluorescence intensity for each DPR translated from the C_4G_2 antisense strand in HEK293T cells transiently co-expressing an NES-mIFP-mIFP-NES fusion protein cytoplasmic marker.

D C_4G_2-Dendra2 non-AUG-dependent fluorescent reporter constructs show cell-type variability in the number of cells that are DPR positive and the overall DPR levels. Transient cotransfections of HEK293T cells robustly undergo C_4G_2-Dendra2 non-AUG-dependent translation compared to NSC34 or rat primary cortical neurons. DPR-positive cells and DPR fluorescent intensity were analyzed and calculated 24 h post-transfection only in cells expressing the cotransfected NES-mIFP construct. HEK293T: $n = 5$ with > 500 cells analyzed per experiment per condition. NSC34: $n = 5$ with $m > 500$ cells per n. Rat primary cortical neuron: $n = 5$ with $m > 40$ cells per n. ***$P < 0.001$. Statistical comparisons were performed using a *t*-test with error bars representing the mean ± SEM.

E Plasmid fluorescent reporter constructs developed for transfection marker and AUG-dependent translation levels. Nuclear export tags drive the fluorescent reporter to highlight cytoplasm over nucleus aiding localization analysis.

F Dot blot analysis performed on lysates of HEK293T containing all sense-ORF reporter constructs. All sense DPR species are detected through DPR-specific antibodies regardless of the ORF of the Dendra2 and HA tags. Additionally, immunofluorescence analysis performed in HEK293T cells containing the sense-ORF reporter constructs demonstrates that Dendra2 is being translated in frame of the correct DPR. DPR-specific antibody staining for the specific ORF reporter construct colocalizes with the Dendra2 tag in frame of the corresponding DPR.

G qRT–PCR analysis measuring transcript levels of C9 reporter constructs. In HEK293T, GA demonstrates higher transcript levels, followed by GR then GP. NSC34 demonstrate higher transcript levels than HEK293T. Conditions with stress, or stress with therapeutic inhibitors, demonstrate varying transcript levels around the same C_T value. Transcript levels have no correlation to the level of RAN-translated products measured. Dendra2 CT values were normalized to NES-mIFP C_T values to account for potential translation efficiency differences between experimental designs.

Source data are available online for this figure.

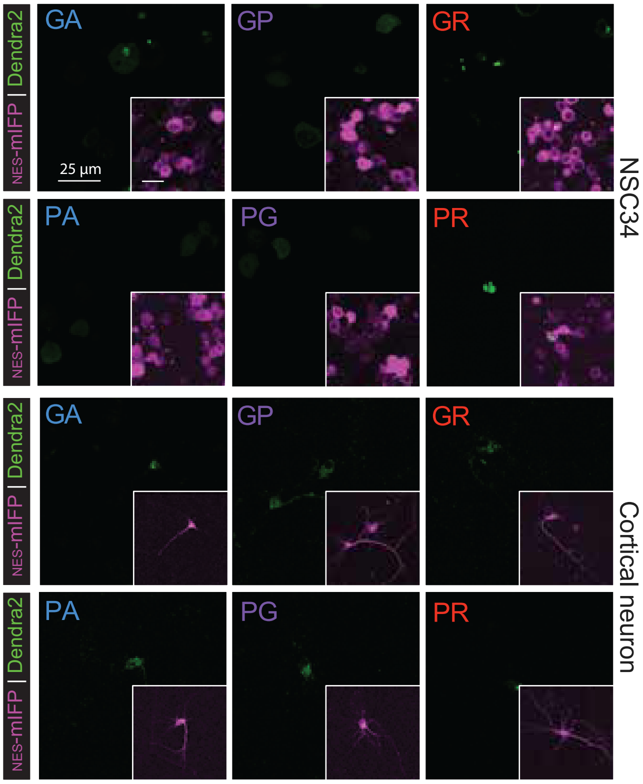

Figure EV2. Localization patterns of non-AUG translated DPRs in NSC34 and primary cortical neurons are similar to patient DPR pathology.

Fluorescent microscopy images of the *C9orf72* NRE non-AUG-dependent reporter constructs show recapitulate localization patterns in NSC34 and cortical neurons as seen in HEK293T models. Both DPR positive and fluorescent intensity levels are lower in NSC34 and rat primary cortical neurons in contrast to HEK293T cells.

© 2019 The Authors

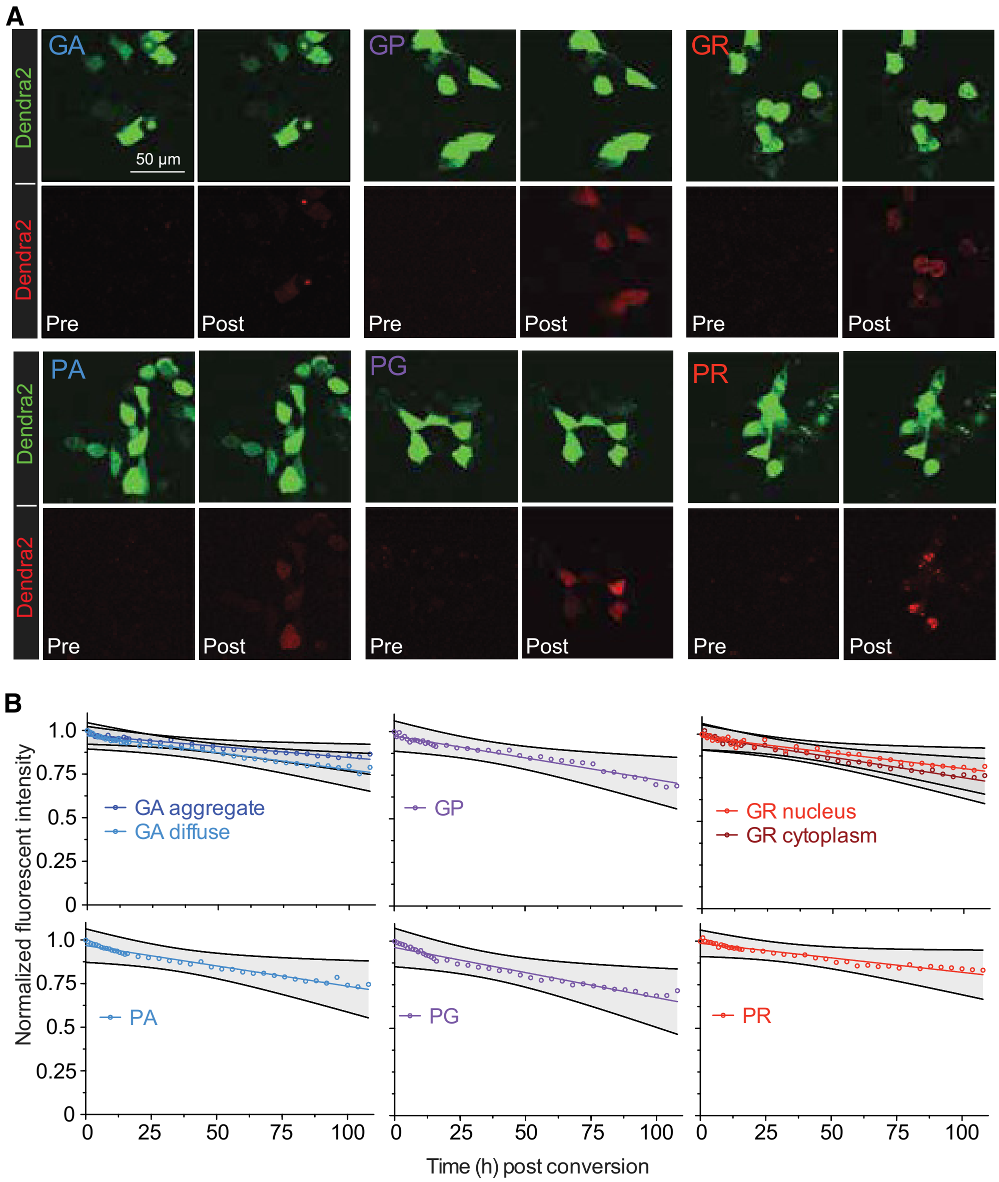

Figure EV3. Photoconversion of the Dendra2 DPR tag generates a red fluorescent DPR that can be longitudinally monitored and demonstrates DPRs have long half-lives.

A The Dendra2 tag in the *C9orf72* NRE non-AUG-dependent reporter constructs is photoconvertible from Dendra2-green to Dendra2-red using successive 405 nm irradiations for 15s. Representative images demonstrate pre- and post-photoconversion of each G_4C_2 or C_4G_2 DPR produced through different ORFs in the *C9orf72* NRE non-AUG-dependent reporter. Photoconverted DPRs retain localization patterns seen from unconverted counterparts.

B Half-lives of photoconverted DPRs produced through the *C9orf72* NRE non-AUG-dependent reporter can be determined by monitoring decreased red fluorescent intensity. Fluorescence levels of photoconverted DPRs were followed for a total of 108 h. GA diffuse and GA aggregates were monitored separately. $n = 3$, with $m > 10$ cells measured per n. The longitudinal fluorescent intensity quantification of photoconverted DPRs produced through the *C9orf72* NRE non-AUG-dependent translation demonstrates long half-lives. Quantification was performed over a total of 108 h. GA diffuse and GA aggregates were measured separately. GR cytoplasm and GR nucleus were measured separately. $n = 3$ with $m > 10$ cells per n. See also Table EV1. Grey shaded regions marked by black lines define the 95% confidence interval.

Source data are available online for this figure.

Figure EV4. Dose–response curves demonstrate increases in non-AUG translation from a spectrum of cellular stressors.

The number of fluorescent-based DPR-positive cells is increased in a dose-dependent manner during cellular stress. Non-AUG-dependent DPR translation was induced using various compounds that produced different types of cellular stress (green = ER stress; blue = oxidative stress; red = multiple stress targets; orange = nuclear export inhibition; tan = HDAC inhibitor; purple = excitotoxic stressors). Compounds were added to cells in a logarithmic dose, and the non-AUG-dependent translation of the *C9orf72* DPR fluorescent reporter was monitored per cell; a DPR-positive cell shows Dendra2 fluorescence signal. Compounds were compared to the vehicle they were dissolved in, either PBS or DMSO. The C9 DPR reporter in the GP frame was utilized for these experiments. Statistical comparisons were performed using *t*-tests per concentration. EC50 values and the usage of these compounds in further experiments are shown in Table EV2. NSC34: $n = 3$ with > 500 cells analyzed per experiment per condition. ***$P < 0.001$, **$P < 0.01$, *$P < 0.05$. Error bars represent the mean ± SEM.

© 2019 The Authors

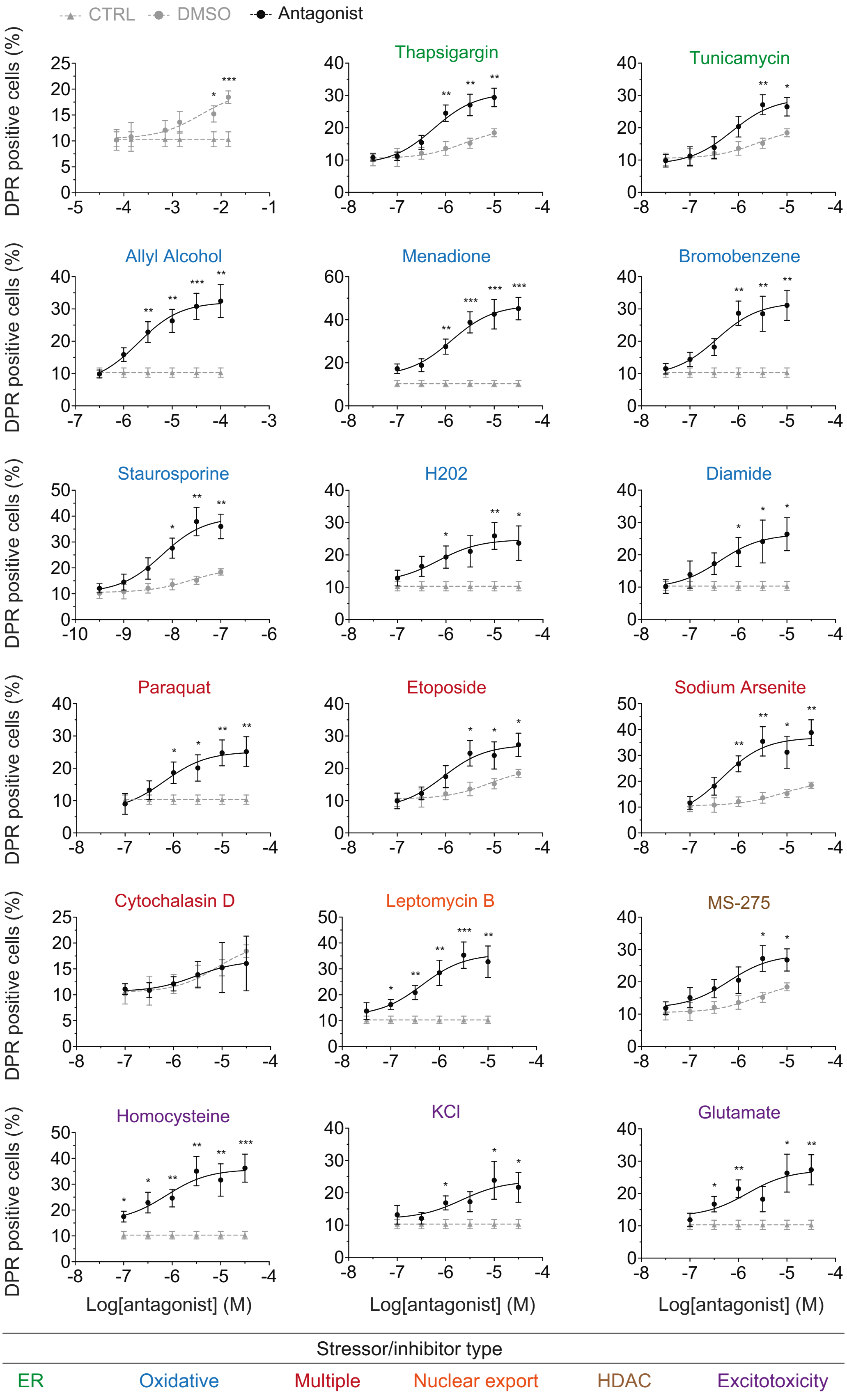
CTRL
DMSO
Antagonist
Thapsigargin
Tunicamycin
Allyl Alcohol
Menadione
Bromobenzene
Staurosporine
H202
Diamide
Paraquat
Etoposide
Sodium Arsenite
Cytochalasin D
Leptomycin B
MS-275
Homocysteine
KCl
Glutamate
DPR positive cells (%)
Log[antagonist] (M)
Stressor/inhibitor type
ER
Oxidative
Multiple
Nuclear export
HDAC
Excitotoxicity

Figure EV4.

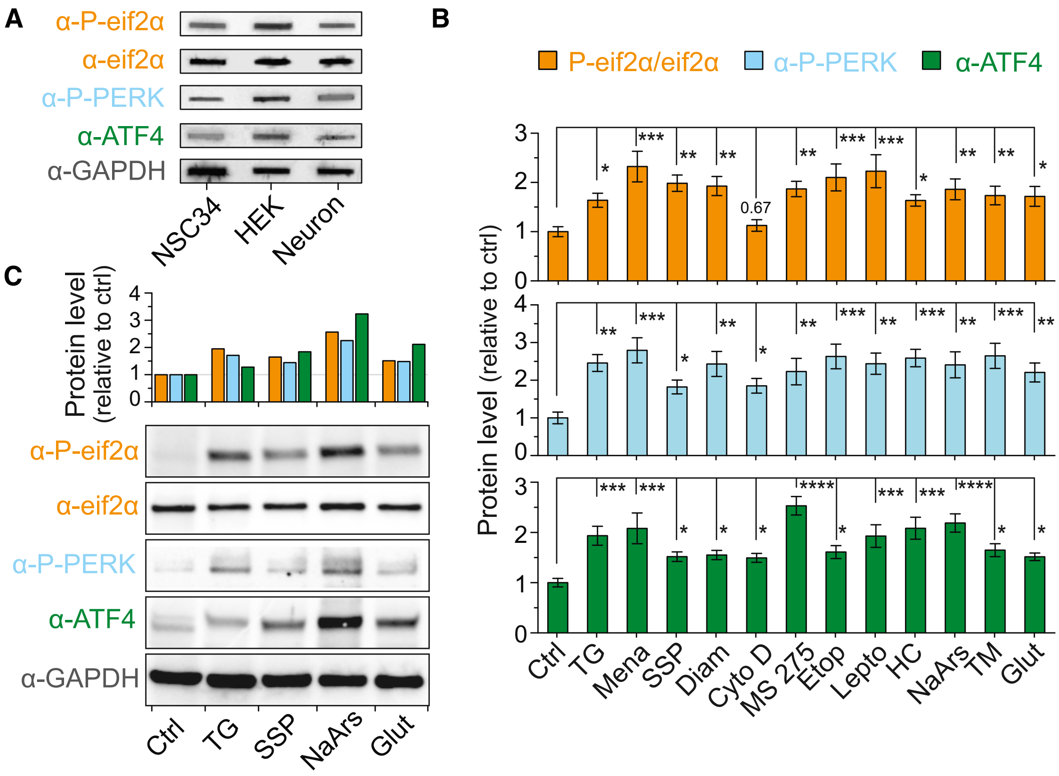

Figure EV5. Basal cell-type differences of ISR protein levels and increases in ISR proteins are concomitant with DPR-inducing stressors.

A Filter-trap binding assays show that the ISR proteins are more active in HEK293T cells versus NSC34 and cortical neurons. These basal ISR levels correlate with DPR levels produced in basal conditions. $n = 4$.

B All stressors increase ISR proteins fluorescent intensity levels in iPSC sMNs. These values are identical to those seen in Fig 5A, but each protein quantification is split for individual statistical analysis using one-way ANOVA. Changes in ISR protein levels are shown relative to non-stressed or DMSO-only-treated cells (CTRL) depending on the vehicle compounds were dissolved in. $n = 4$. ****$P < 0.0001$, ***$P < 0.001$, **$P < 0.01$, *$P < 0.05$. Error bars represent the SEM.

C Western blot analysis shows changes in ISR protein levels are consistent with filter-trap binding assay results.

 © 2019 The Authors
