# Supplementary material for: Repeat‐associated non‐AUG translation in C9orf72‐ALS/FTD is driven by neuronal excitation and stress
Source: EMBO Mol Med. 2019 Jan 7;11(2):e9423. doi: 10.15252/emmm.201809423 (PMC6365928; doi:10.15252/emmm.201809423)
Supplement: Supplementary file 3 — Table EV1 [file EMMM-11-e9423-s003.pdf]

Condition	Slope	Y-intercept	Equation (y = value related to max; x = time)	Actual Value @ 108hrs	Predicted Value @ 108 hrs	Predicted Half-Life (value=0.5)
GA Diffuse	-0.00194	0.9698	y = -0.00194x + 0.9698	0.789	0.760	242.16 hrs
GA Aggregate	-0.00127	0.9724	y = -0.00127x + 0.9724	0.865	0.835	371.97 hrs
GP	-0.0025	0.9751	y = -0.00250x + 0.9751	0.689	0.705	190.04 hrs
GR Cytoplasm	-0.00235	0.9855	y = -0.00235x + 0.9855	0.763	0.732	206.60 hrs
GR Nucleus	-0.00171	0.9827	y = -0.00171x + 0.9827	0.815	0.798	282.28 hrs
PA	-0.00232	0.9683	y = -0.00232x + 0.9683	0.746	0.718	201.85 hrs
PG	-0.00287	0.9639	y = -0.00287x + 0.9639	0.716	0.654	161.64 hrs
PR	-0.00166	0.9881	y = -0.00166x + 0.9881	0.834	0.809	294.04 hrs

Table EV1. Calculations of DPR half-life using longitudinal fluorescent imaging quantification of photoconverted DPR decay over 108 hrs.

Quantification of photoconverted DPR fluorescent intensity was plotted over 108 hrs (see Figure EV5). The slope and y-intercept for each conditioned was calculated and used to generated a slope-intercept form equation for each condition. The equation was validated for accuracy by comparing predicted value at 108 hrs to the actual value measured. Through this equation, a predicted half-life was calculated by setting fluorescent intensity level (y) to 0.5. Predicted half-life values demonstrate long lived DPRs that are dependent on localization and granularity of each species.
