# Supplementary material for: Repeat‐associated non‐AUG translation in C9orf72‐ALS/FTD is driven by neuronal excitation and stress
Source: EMBO Mol Med. 2019 Jan 7;11(2):e9423. doi: 10.15252/emmm.201809423 (PMC6365928; doi:10.15252/emmm.201809423)
Supplement: Supplementary file 4 — Table EV2 [file EMMM-11-e9423-s004.pdf]

Stressor	Stress Type	DPR50 (μM)	Used in Future Experiments	Notes
Thapsigargin	ER stress	0.960	Y – 1 μM	in DMSO
Tunicamycin	ER stress	1.340	Y – 1 μM	in DMSO
Allyl Alcohol	Oxidative Stress	2.990	N	Highly toxic at higher doses; higher fluorescence background
Menadione	Oxidative Stress	2.310	Y – 2 μM	Toxic at higher doses; high fluorescence background; reliable induction of RANT
Bromobenzene	Oxidative Stress	3.570	N	Induction of RAN translation highly variable, not reliable
Staurosporine	Oxidative Stress	0.008	Y – .01 μM	in DMSO
Diamide	Oxidative Stress	3.070	Y – 3 μM	in DMSO
H202	Oxidative Stress	6.810	N	
MS-275	HDAC inhibitor	0.830	Y – 1 μM	in DMSO
Leptomycin B	Nuclear Export Inhibitor	0.640	Y – 1 μM	
Homocysteine	Excitotoxicity/ER stress	0.760	Y – 1 μM	Very reliable induction of RANT
Glutamate	Excitotoxicity	2.570	Y – 5 μM	Very reliable induction of RANT
KCl	Excitotoxicity/Other	2.550	N	Mode of RANT induction unclear (excitotoxic vs other mechanism)
Etoposide	Multiple (ER/Oxidative/Apoptosis)	1.890	Y – 5 μM	in DMSO
Paraquat	Oxidative Stress/Apoptotic	2.530	N	Highly toxic to cells
Cytochalasin D	Cytoskeleton Disruption/Apoptotic	3.850	Y – 5 μM	Toxic at higher doses
Sodium Arsenite	Stress Granule Induction/Oxidative	0.047	Y – .05 μM	in DMSO

Table EV2. Characterization of individual stressors used to increase non-AUG translation and their EC50 values.
A wide range of cellular stressors were applied to NSC34 in a dose dependent manner and assessed for increases in frequency of non-AUG dependent DPR translation (see Figure EV6). Listed is the cellular stressor used, the type of cell stress, and the EC50 value of the increase in frequency of non-AUG translation. Also listed is whether these stressors were utilized in future experiments and well as any notes relevant to the use of the stressor.
