# Supplementary material for: Repeat‐associated non‐AUG translation in C9orf72‐ALS/FTD is driven by neuronal excitation and stress
Source: EMBO Mol Med. 2019 Jan 7;11(2):e9423. doi: 10.15252/emmm.201809423 (PMC6365928; doi:10.15252/emmm.201809423)

Figure 1B and EV1B – Representative Filter Trap Assays

Anti-HA: Figure 1B and EV1B

Order of sample – GA, GP, GR, PA, PG, PR

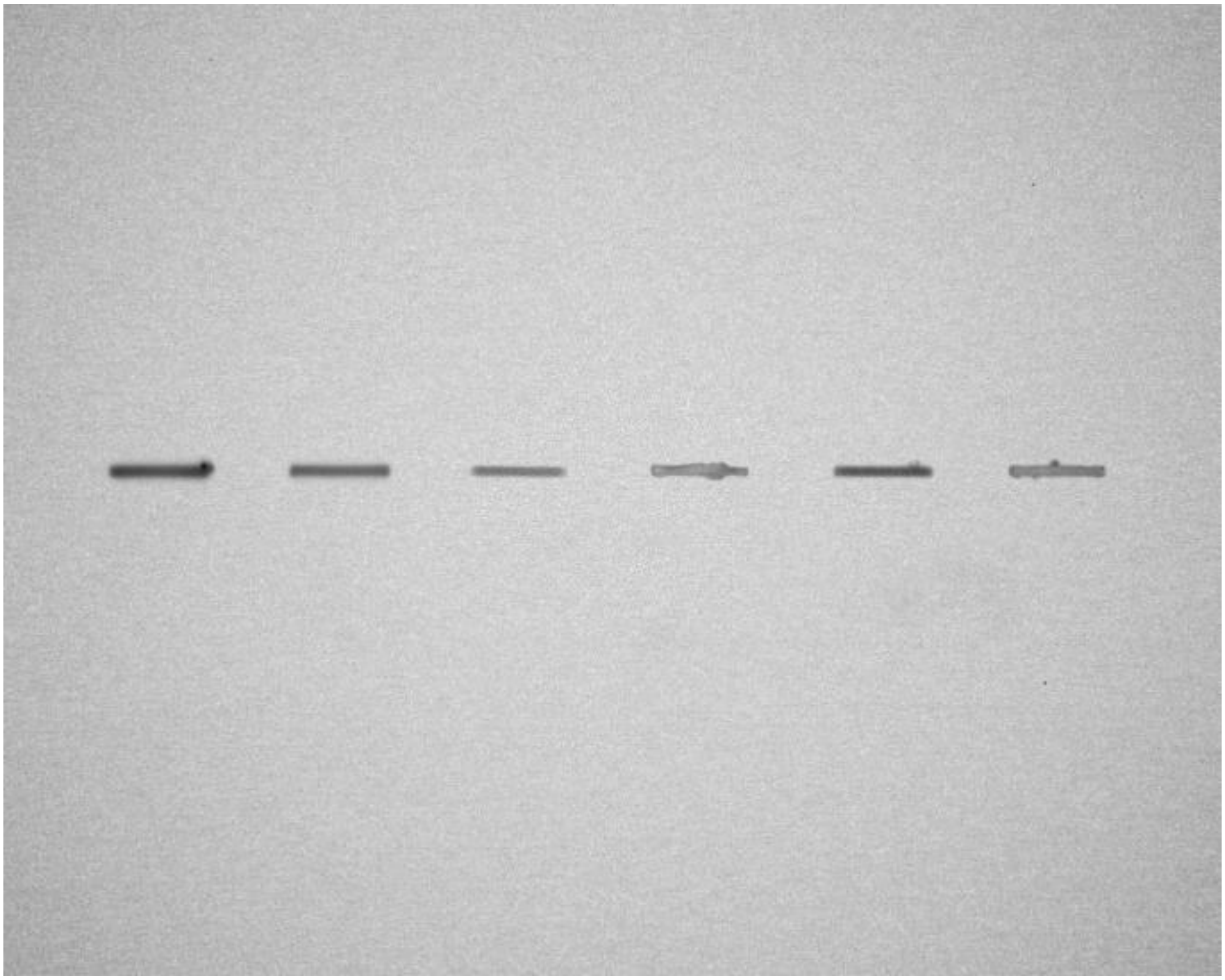

Anti-GAPDH: Figure 1B and EV1B

Same order as above

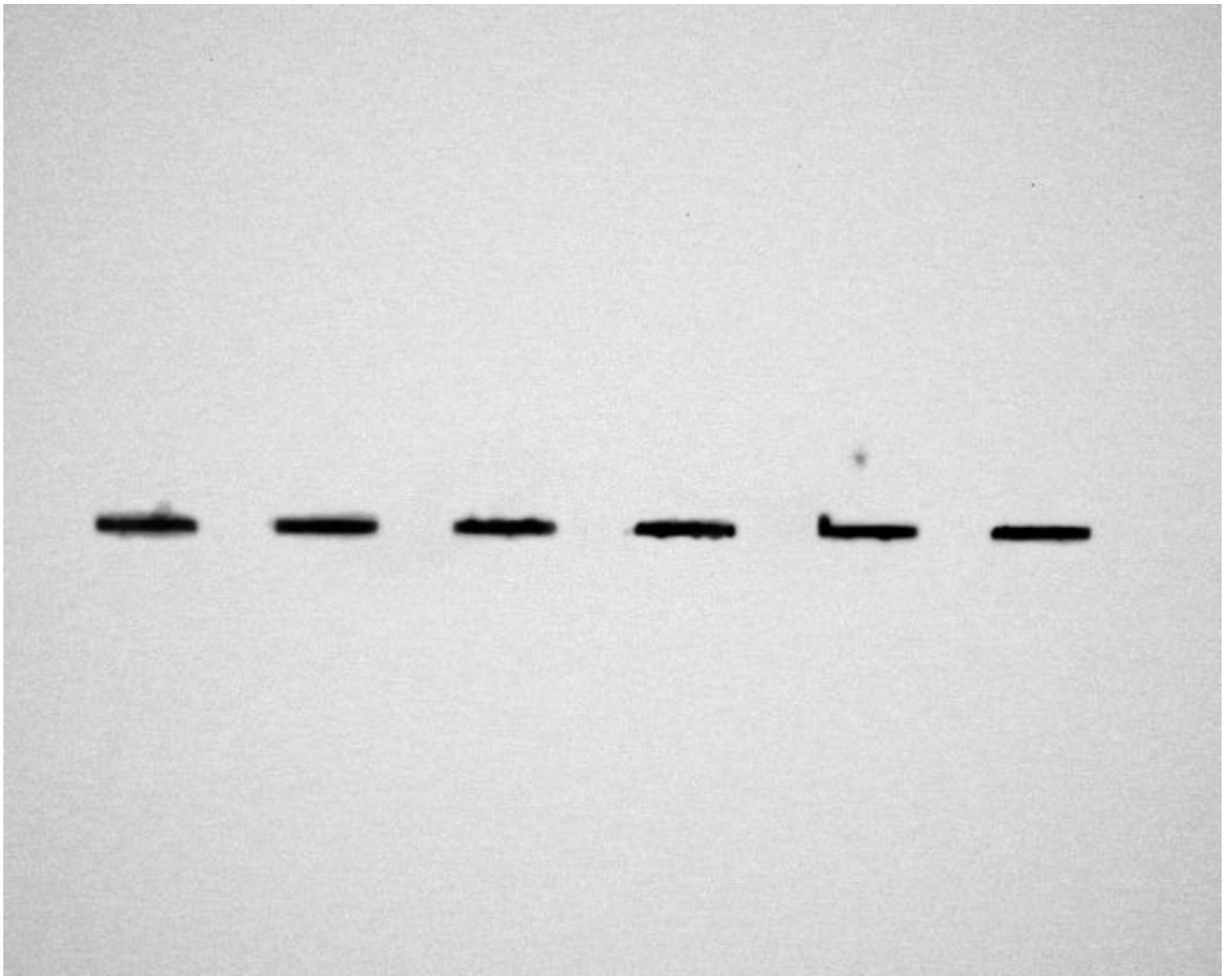

Supplement: Supplementary file 6 — Source Data for Expanded View [file EMMM-11-e9423-s010.zip › emmm201809423-sup-0010-SDataEV/source_data_for_fig_1B_and_EV_1B.pdf]
