# Supplementary material for: Repeat‐associated non‐AUG translation in C9orf72‐ALS/FTD is driven by neuronal excitation and stress
Source: EMBO Mol Med. 2019 Jan 7;11(2):e9423. doi: 10.15252/emmm.201809423 (PMC6365928; doi:10.15252/emmm.201809423)

Figure 5A and EV7A - Representative blots used in figures that have correct order of conditions

FIg 5 - anti-P-eif2α Filter Trap Assay

Line 1 - TG through MS275

Line 2 - Etop through Glut

Line 3 - EV7 HEK, CTRL/ EV7 NSC34, EV7 Neuron

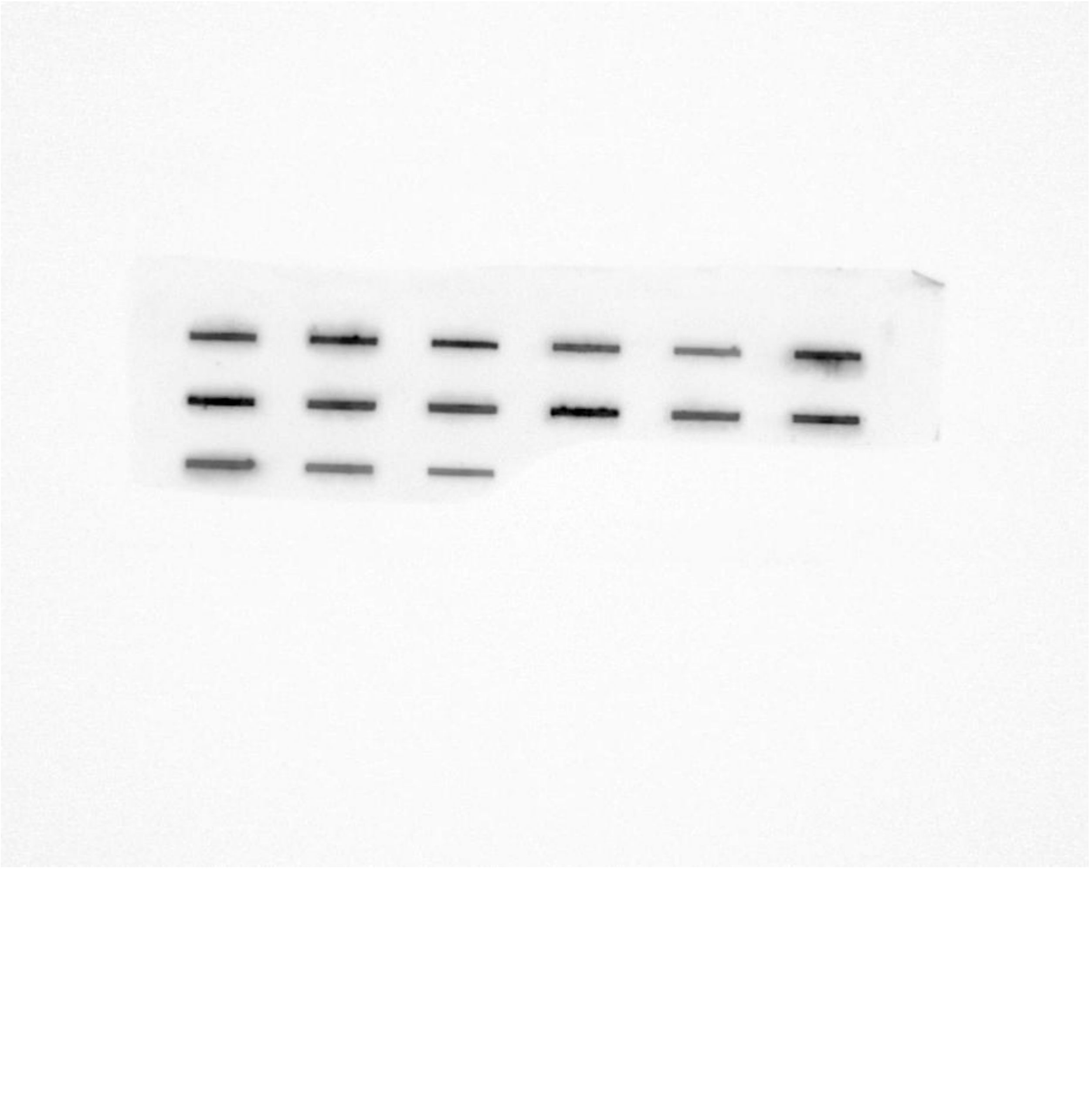

FIg 5 - anti-eif2α Filter Trap Assay

Line 1 - TG through MS275

Line 2 - Etop through Glut

Line 3 - EV7 HEK, CTRL/ EV7 NSC34, EV7 Neuron

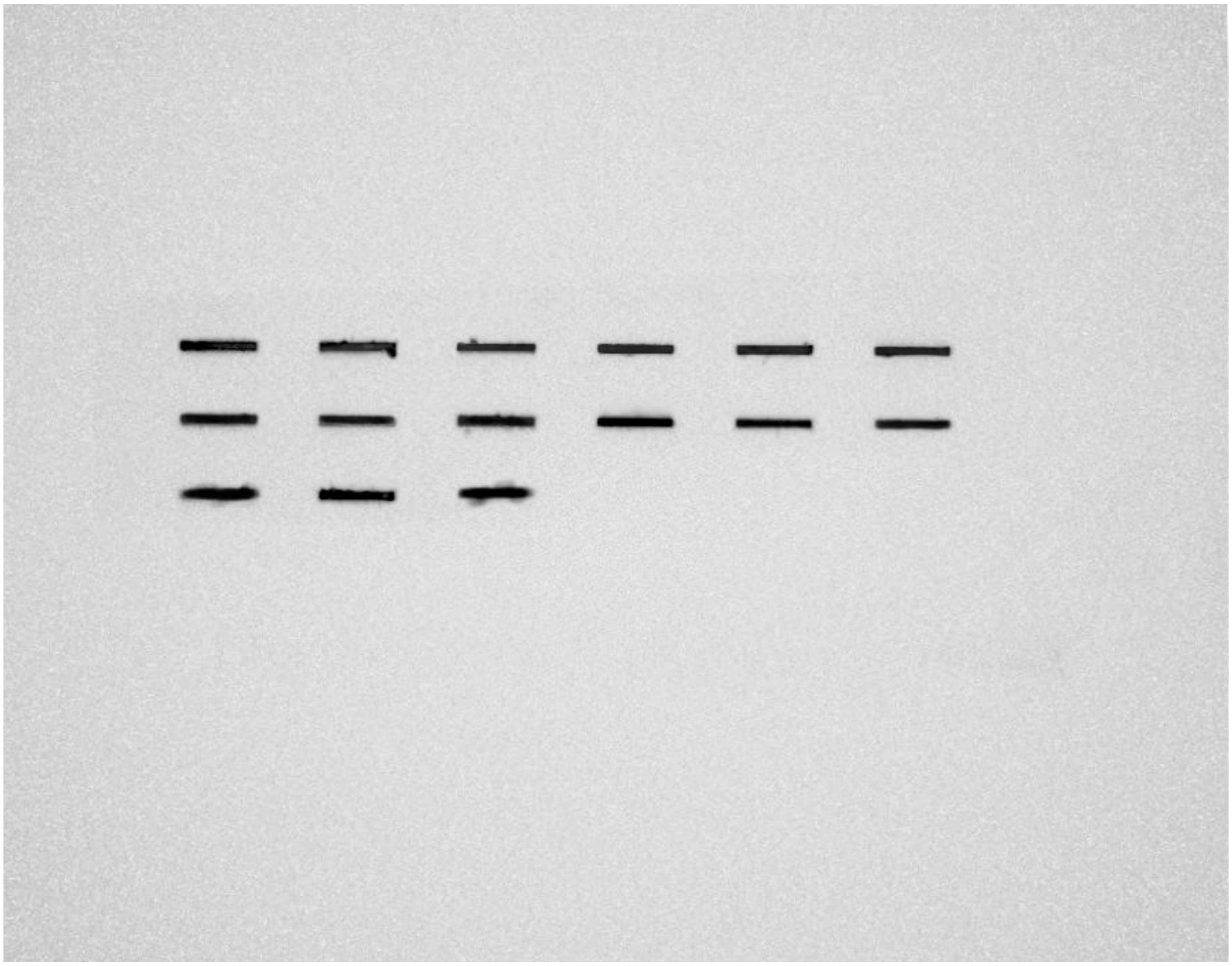

FIg 5 - anti-P-PERK Filter Trap Assay

Line 1 - TG through MS275

Line 2 - Etop through Glut

Line 3 - EV7 Neuron, EV7 HEK, CTRL/ EV7 NSC34

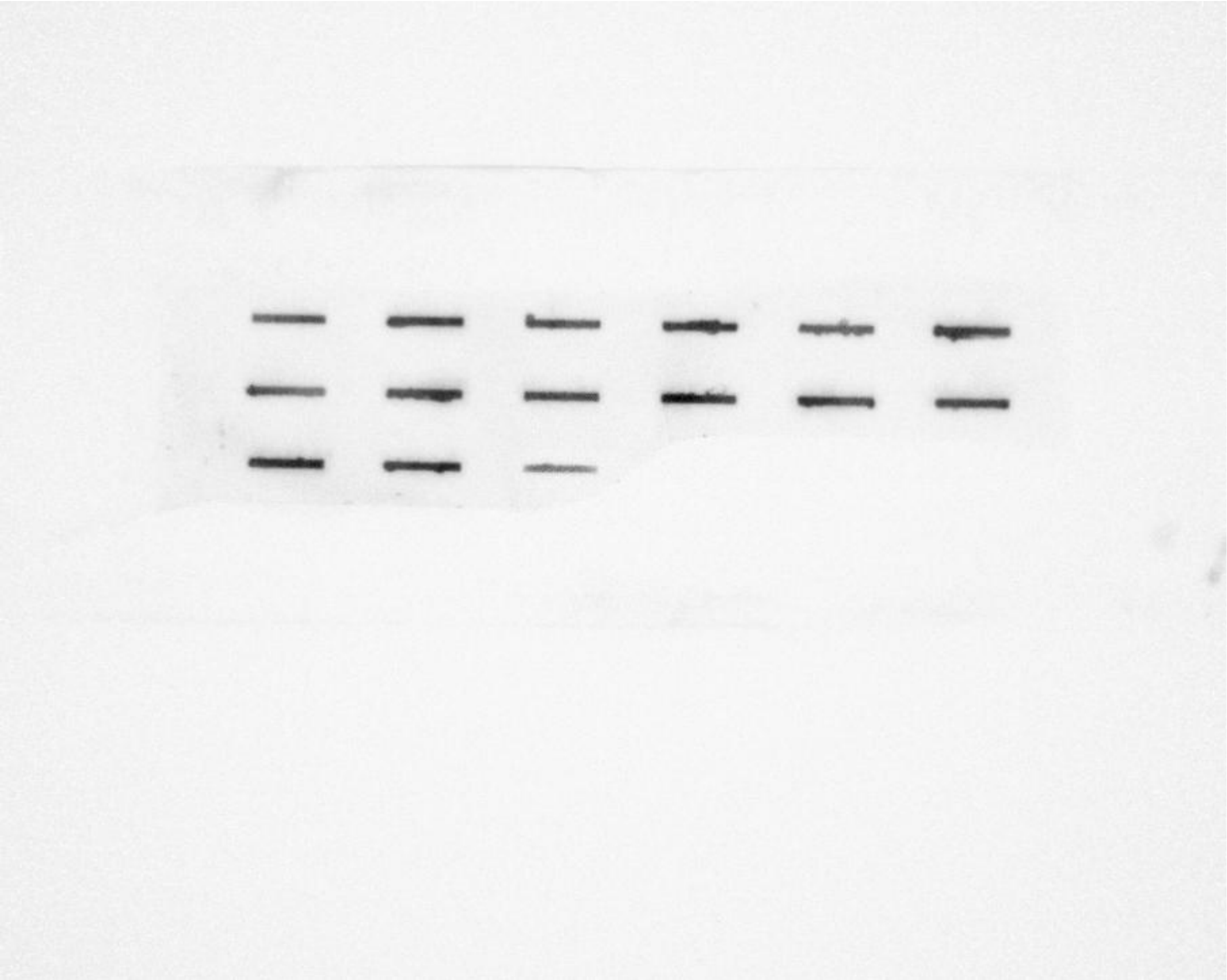

FIg 5 - anti-ATF4 Filter Trap Assay

Line 1 - TG through MS275

Line 2 - Etop through Glut

Line 3 - CTRL/ EV7 NSC34, EV7 HEK, EV7 Neuron

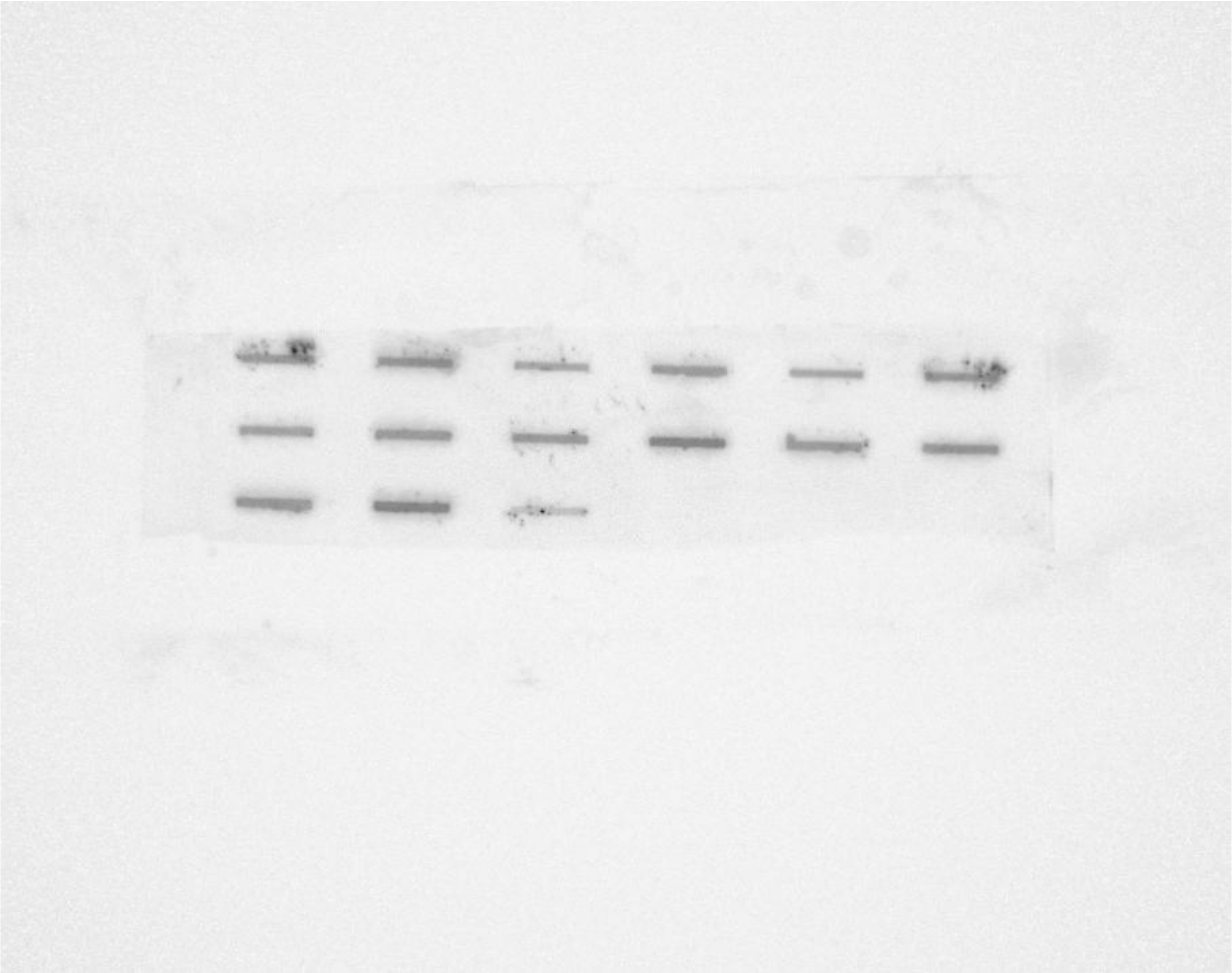

FIg 5 - anti-GAPDH Filter Trap Assay

Line 1 - TG through MS275

Line 2 - Etop through Glut

Line 3 - CTRL/ EV7 NSC34, EV7 HEK, EV7 Neuron

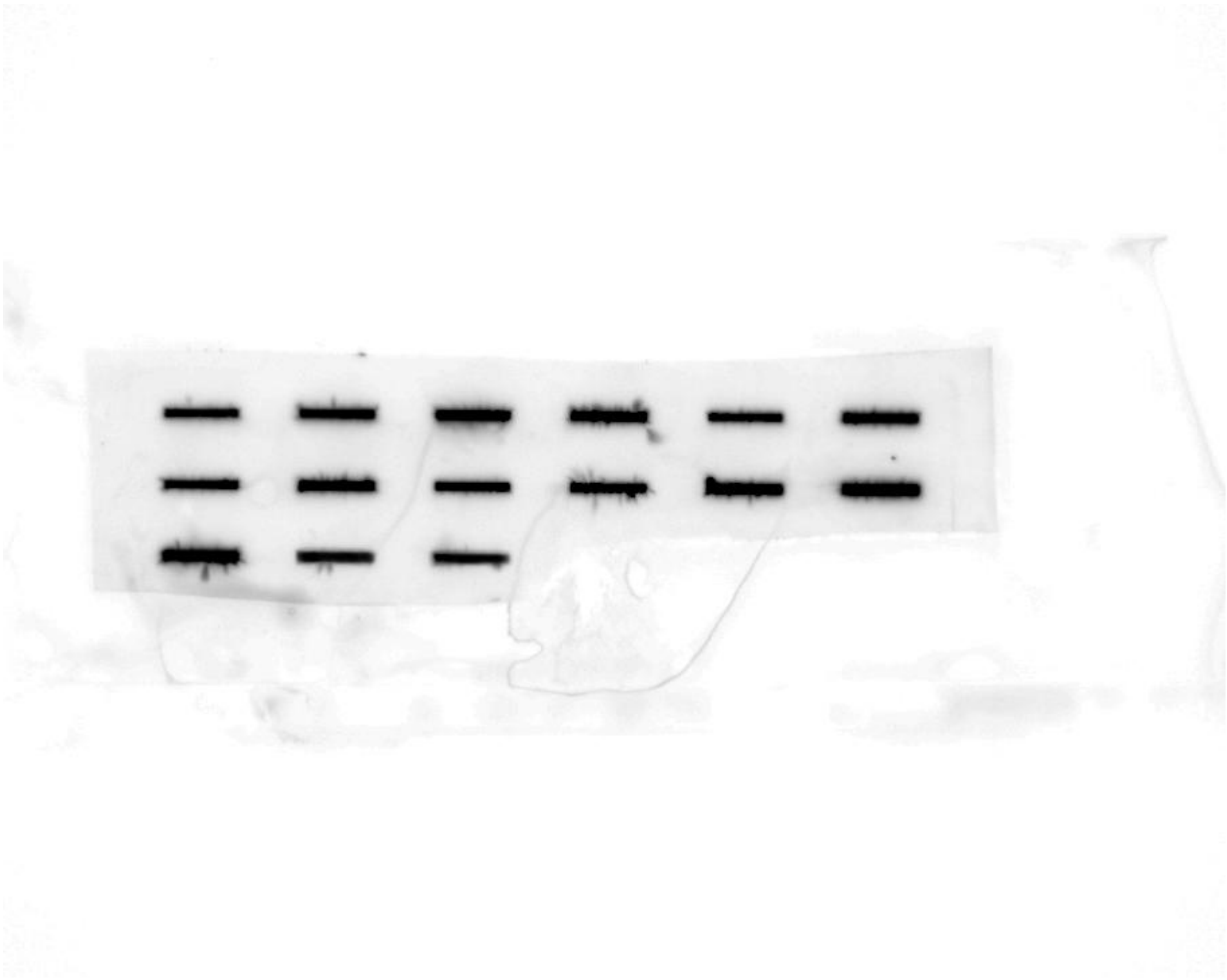

Supplement: Supplementary file 6 — Source Data for Expanded View [file EMMM-11-e9423-s010.zip › emmm201809423-sup-0010-SDataEV/source_data_for_fig_5_and_EV7A.pdf]
