# Supplementary material for: Repeat‐associated non‐AUG translation in C9orf72‐ALS/FTD is driven by neuronal excitation and stress
Source: EMBO Mol Med. 2019 Jan 7;11(2):e9423. doi: 10.15252/emmm.201809423 (PMC6365928; doi:10.15252/emmm.201809423)

Figure 1F – Representative Dot Blot Assays

Anti-GA:

Order – GA-ORF-Dendra2-HA; GP-ORF-Dendra2-HA; GR-ORF-Dendra2-HA

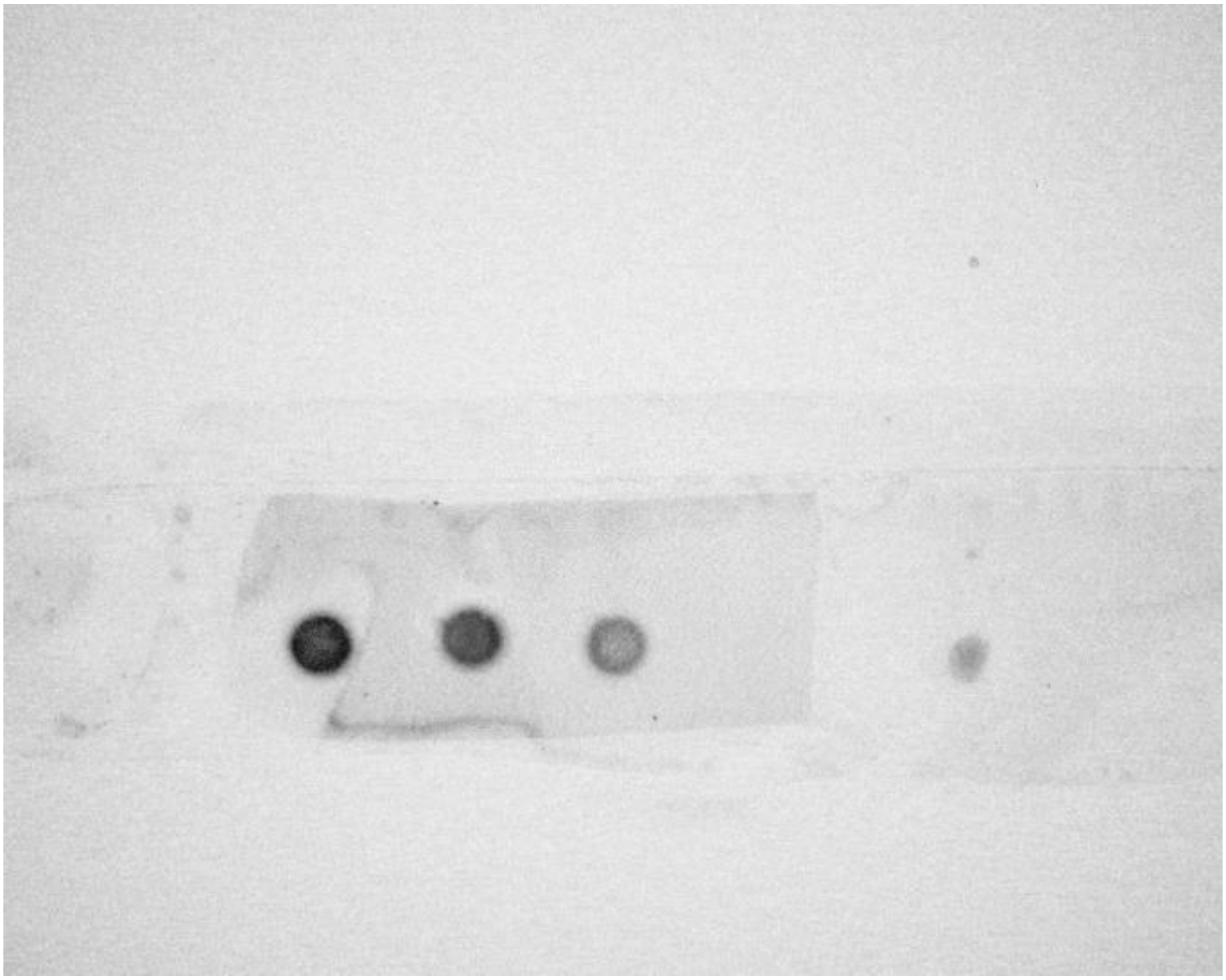

Anti-GP:

Same Order As above

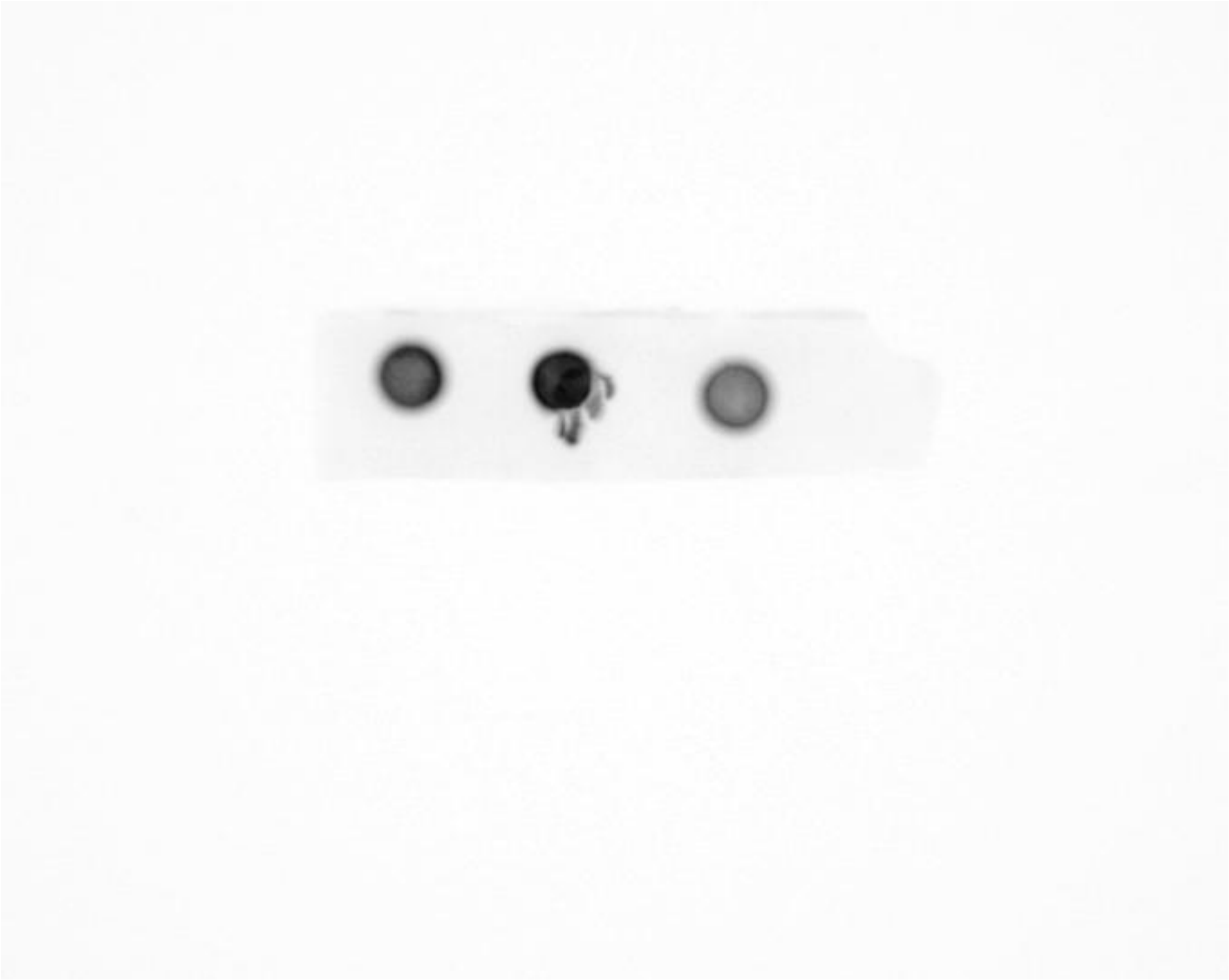

Anti-GR:

Same Order as Above

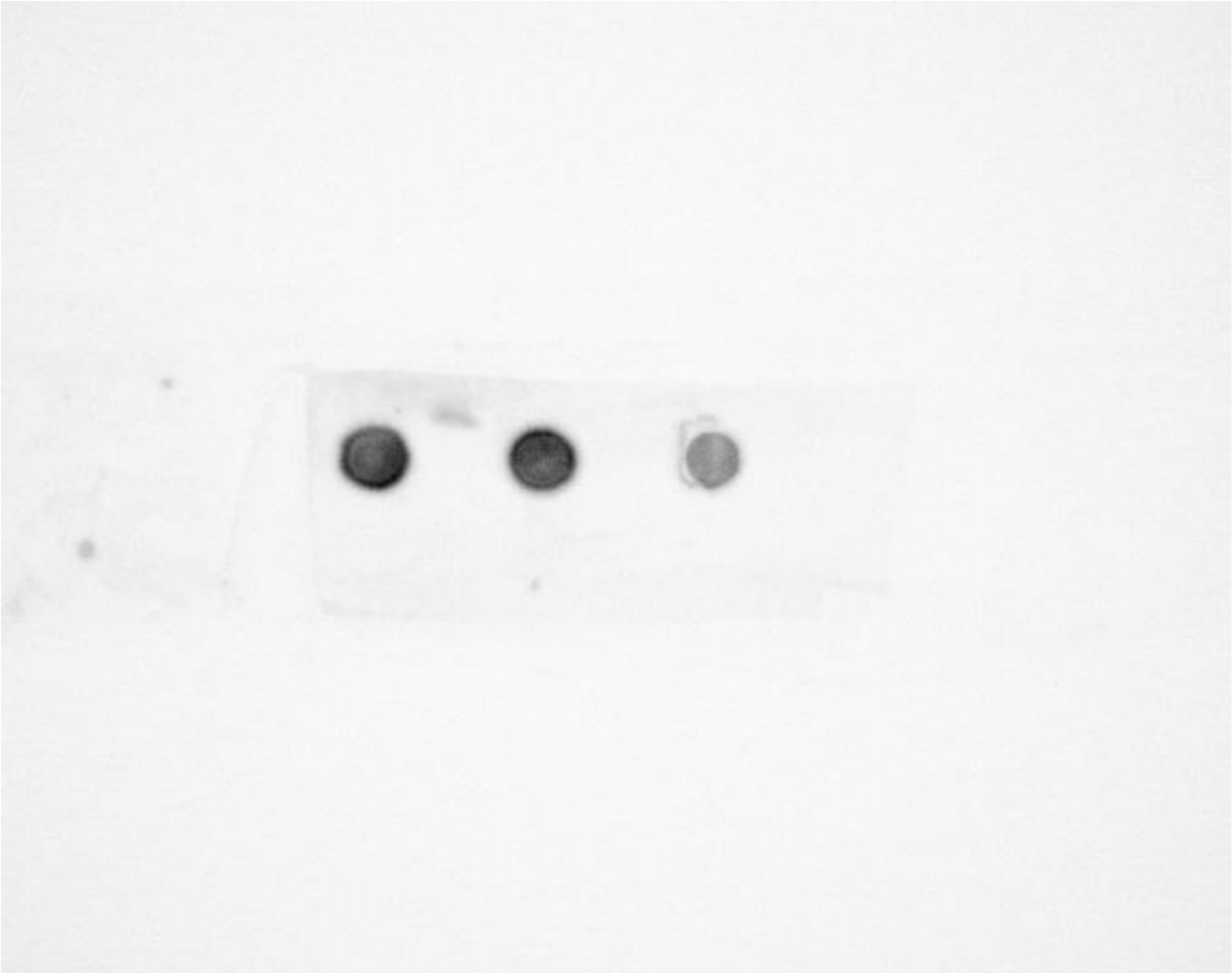

Anti-GAPDH:

Same Order as Above

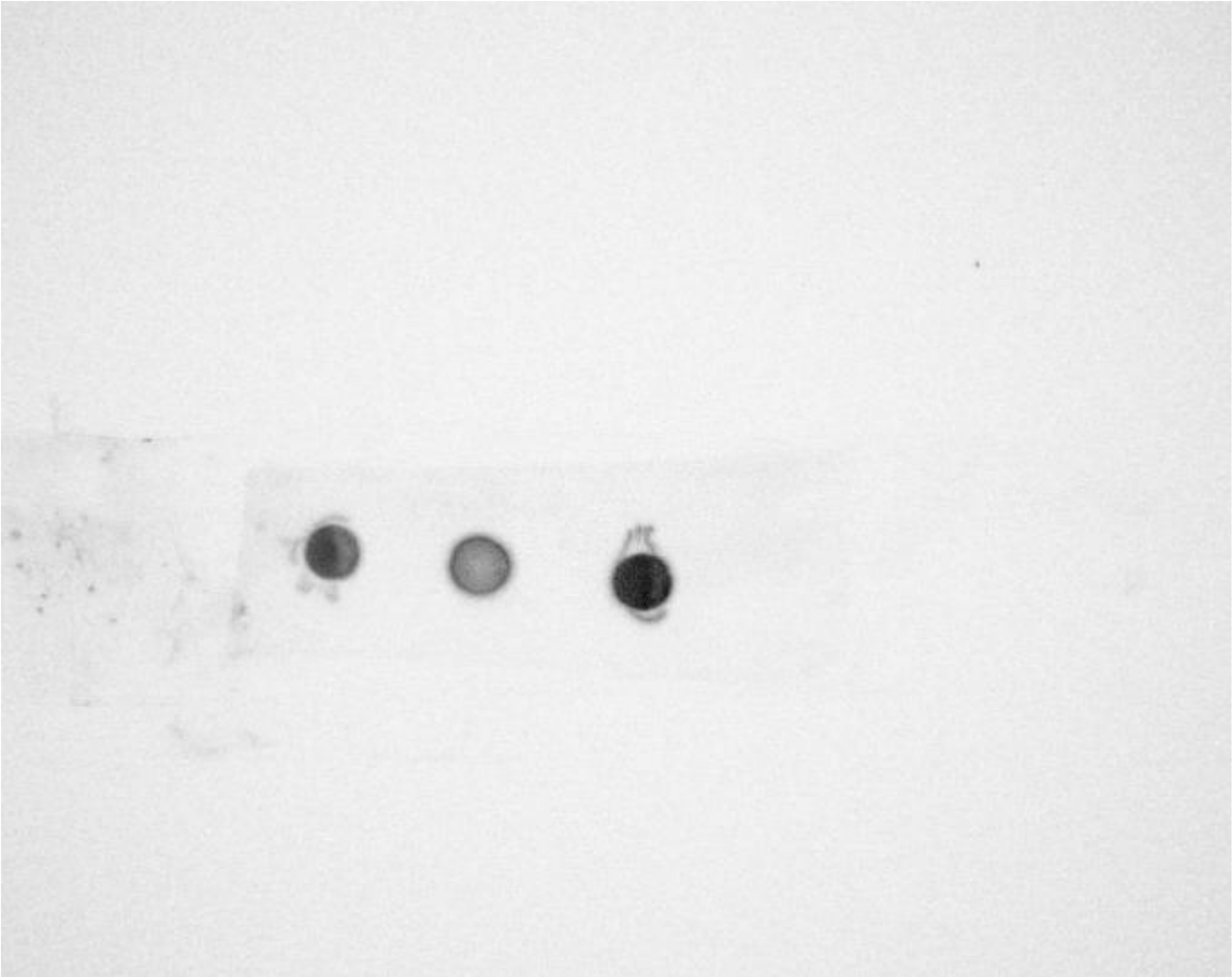

Anti-DPRs in GR-ORF:

Order – Positive CTRL(Not used in Figure, cropped off); Anti-GA; Anti-GP; Anti-GR; CTRL (Negative)

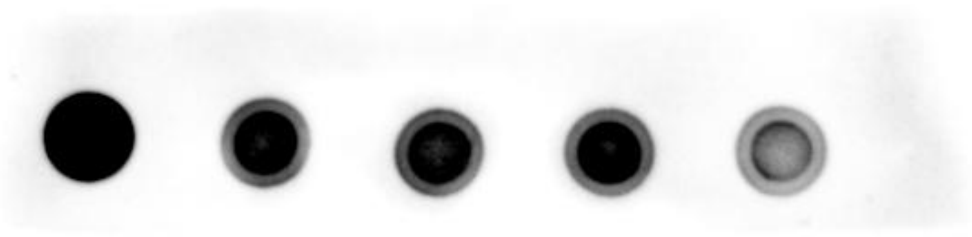

Anti-Actin used for Anti-DPRs above:

Order- Top Row: For a different set of experiments, not used in representative figures but part of same source data

Bottom Row: Positive CTRL(Not used in Figure, cropped off); Anti-GA; Anti-GP; Anti-GR; CTRL (Negative)

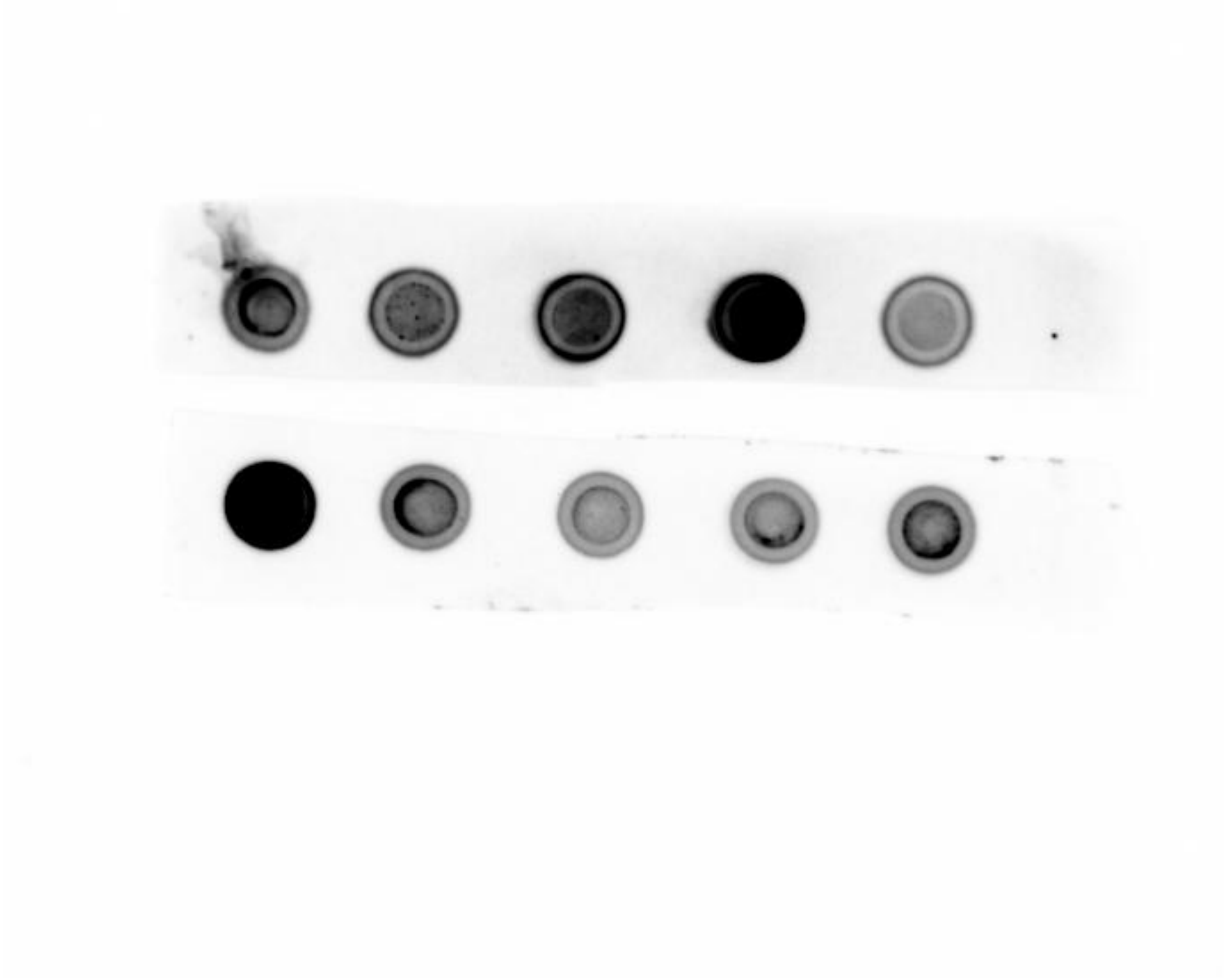

Supplement: Supplementary file 6 — Source Data for Expanded View [file EMMM-11-e9423-s010.zip › emmm201809423-sup-0010-SDataEV/source_data_for_fig_EV1F.pdf]
