# Supplementary material for: Repeat‐associated non‐AUG translation in C9orf72‐ALS/FTD is driven by neuronal excitation and stress
Source: EMBO Mol Med. 2019 Jan 7;11(2):e9423. doi: 10.15252/emmm.201809423 (PMC6365928; doi:10.15252/emmm.201809423)

Figure EV7B - Western Blots

Anti-P-eif2α - lower staining - MW ~38 KDA

Anti-P-PERK - Higher staining - MW ~170 KDA

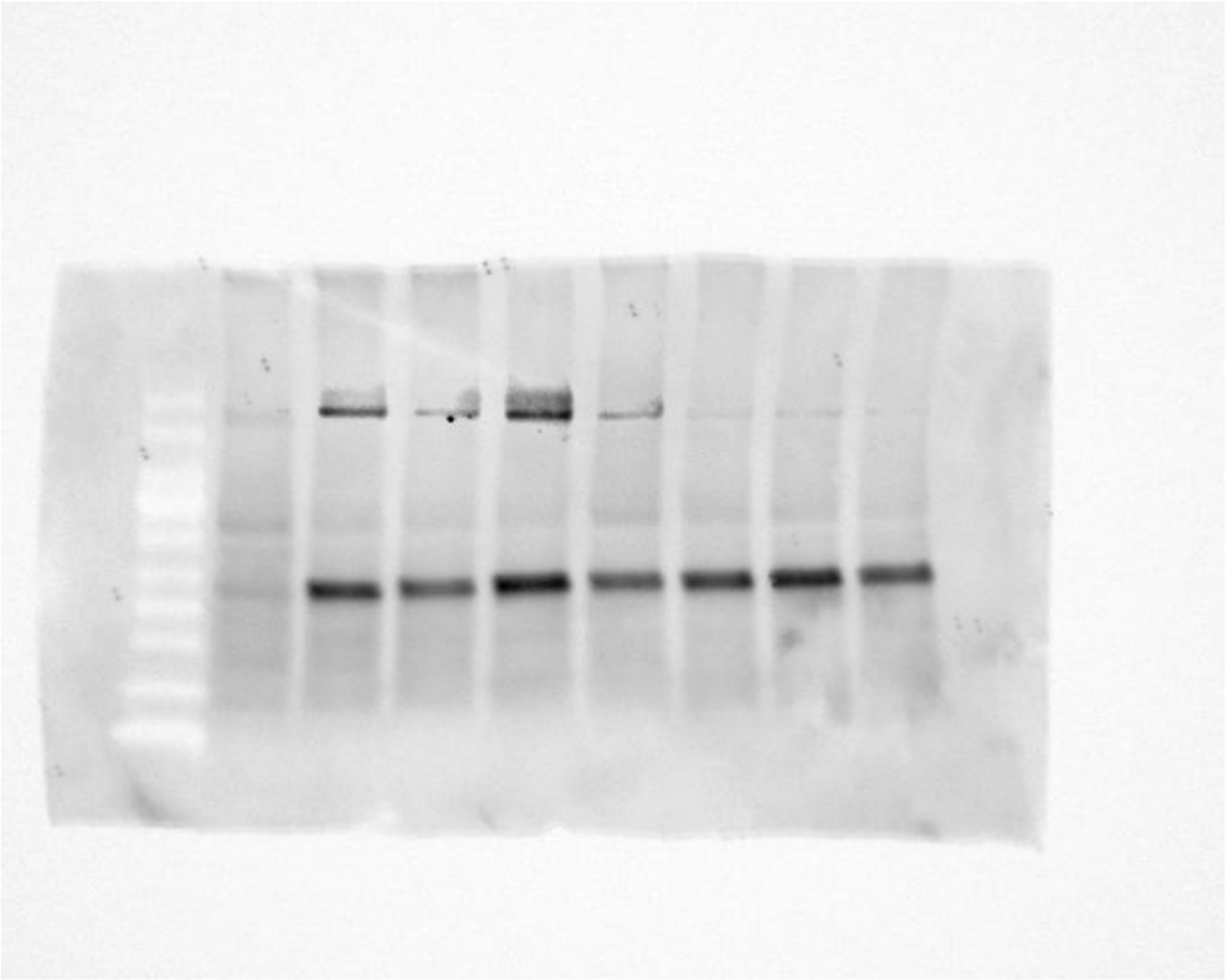

MW marker:

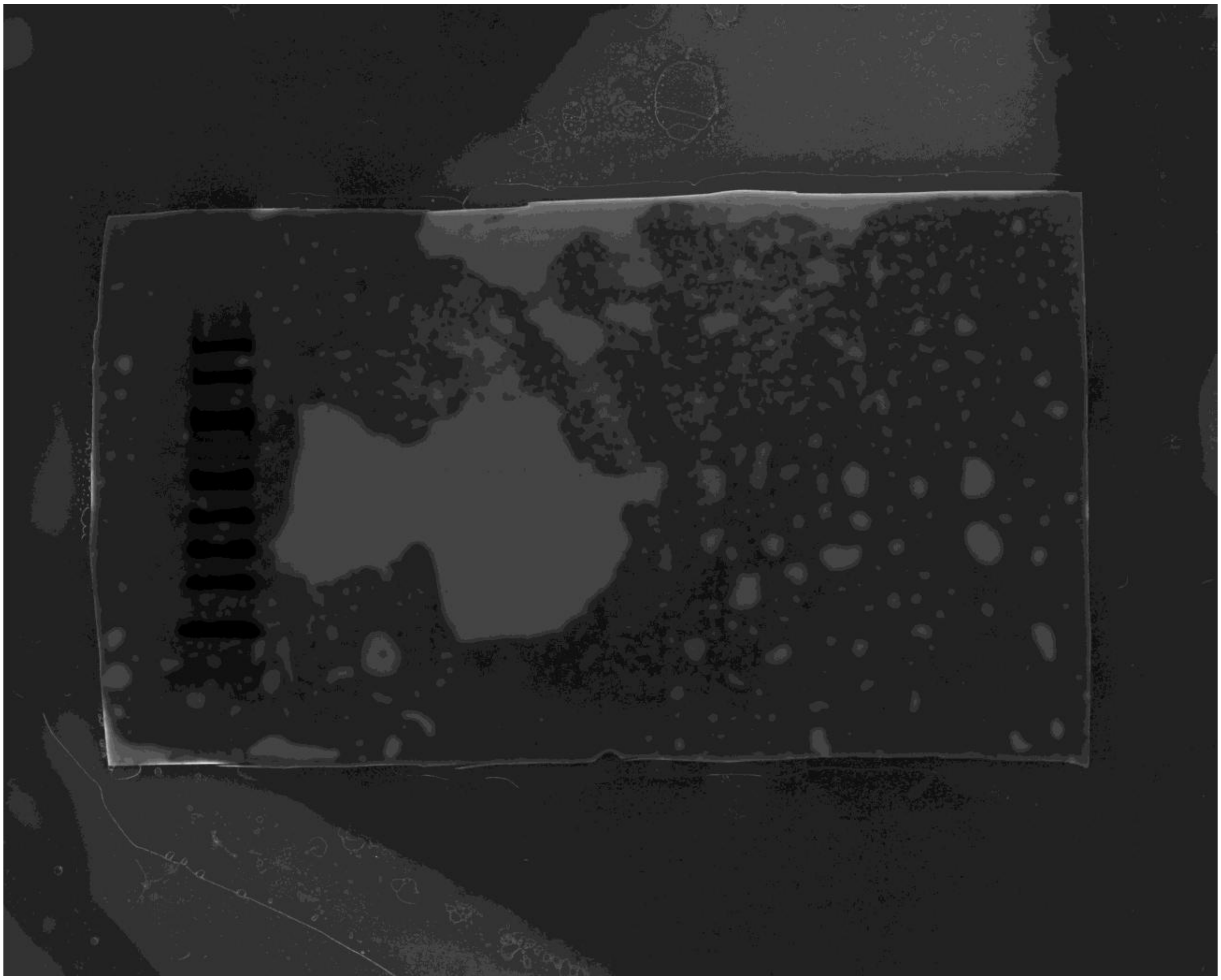

Anti-eif2α:

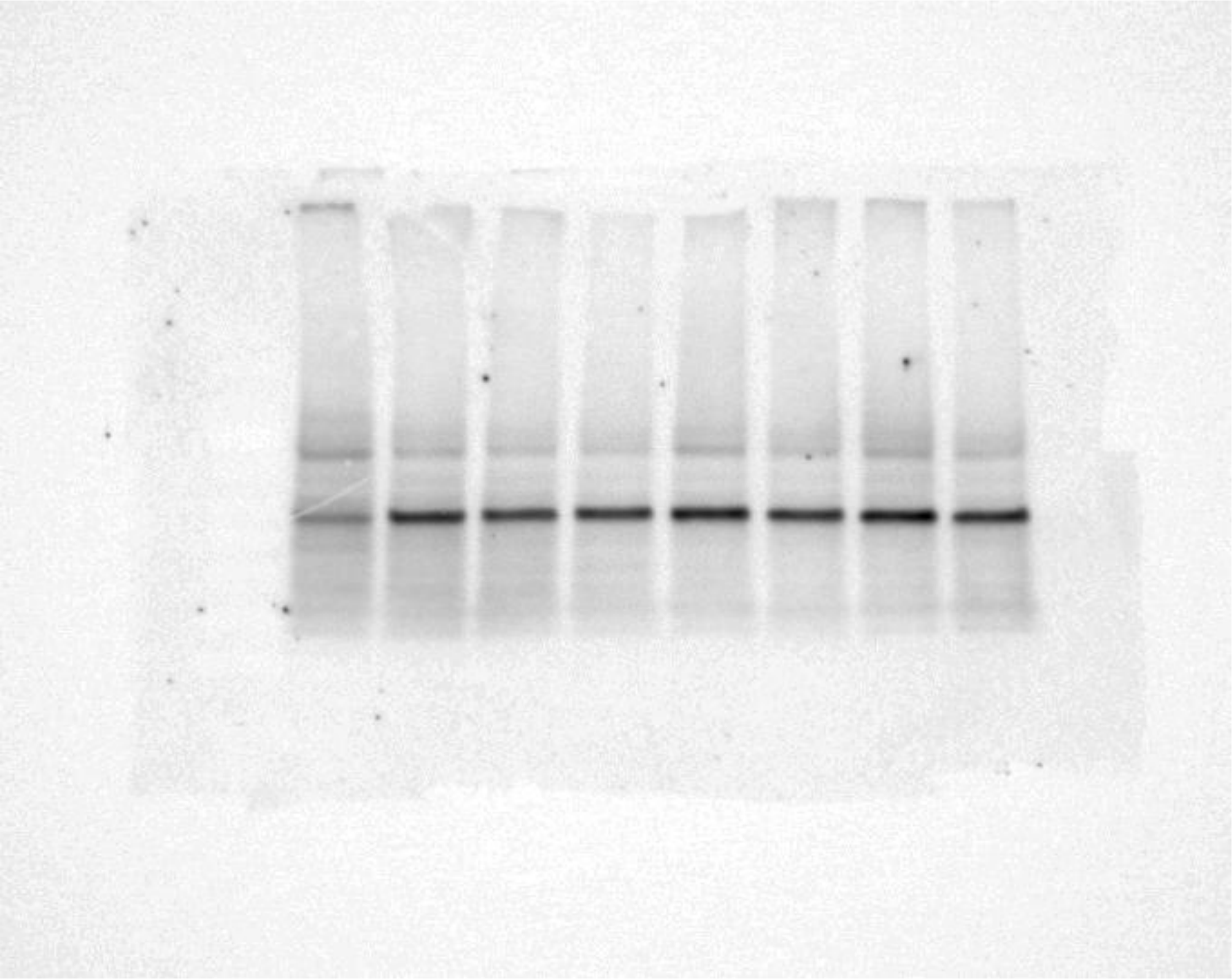

MW Marker :

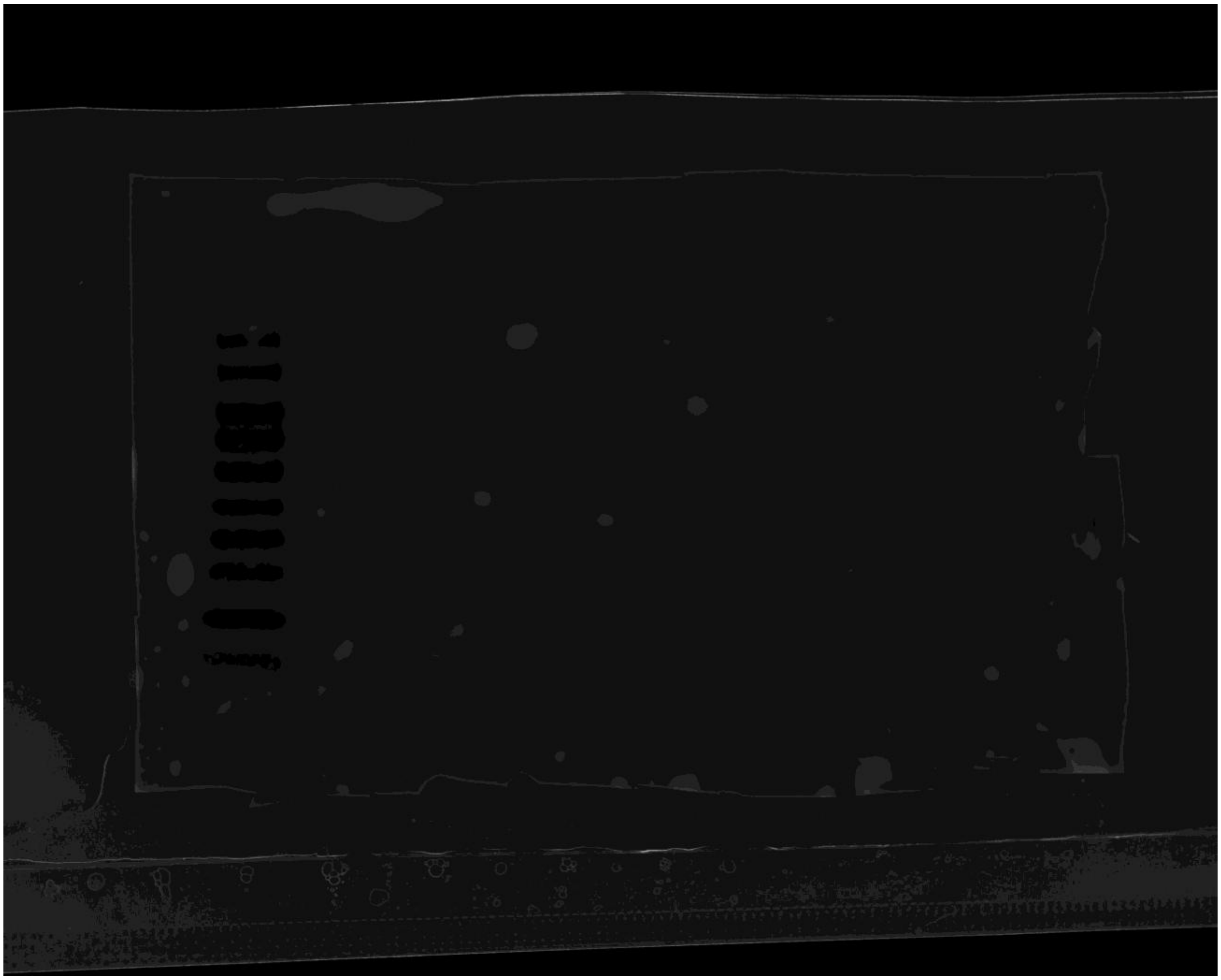

Anti-ATF4:

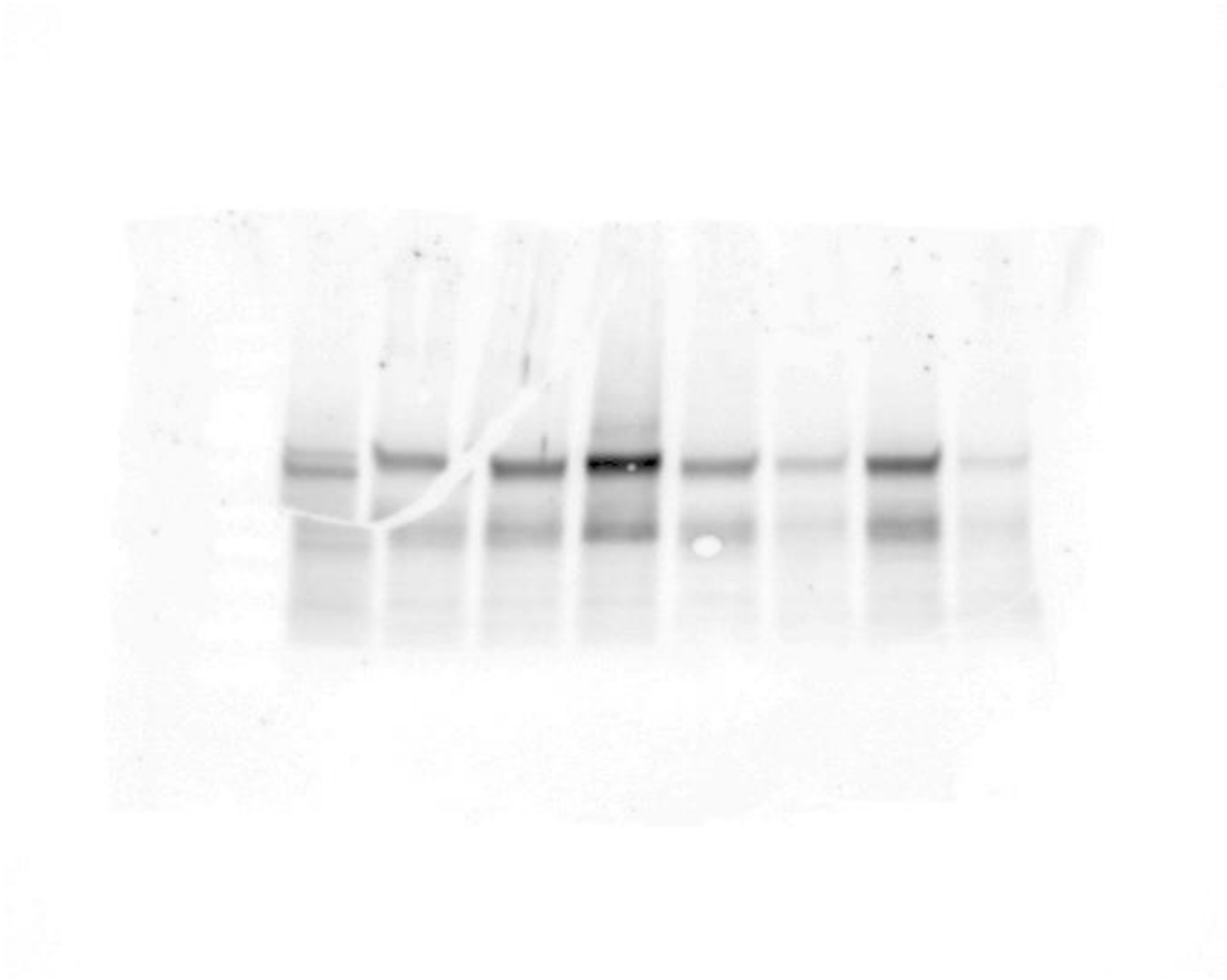

MW Marker:

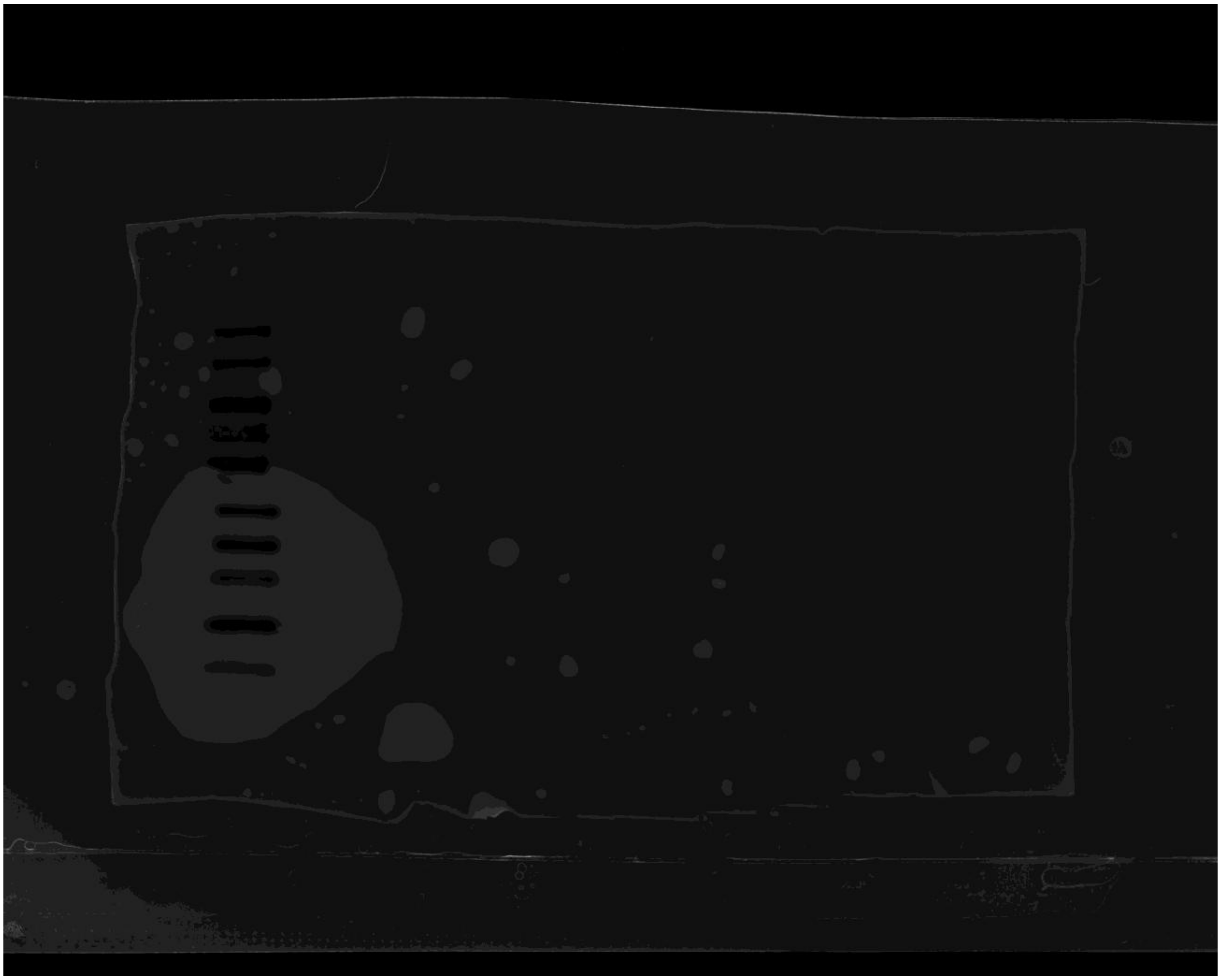

Anti-GAPDH:

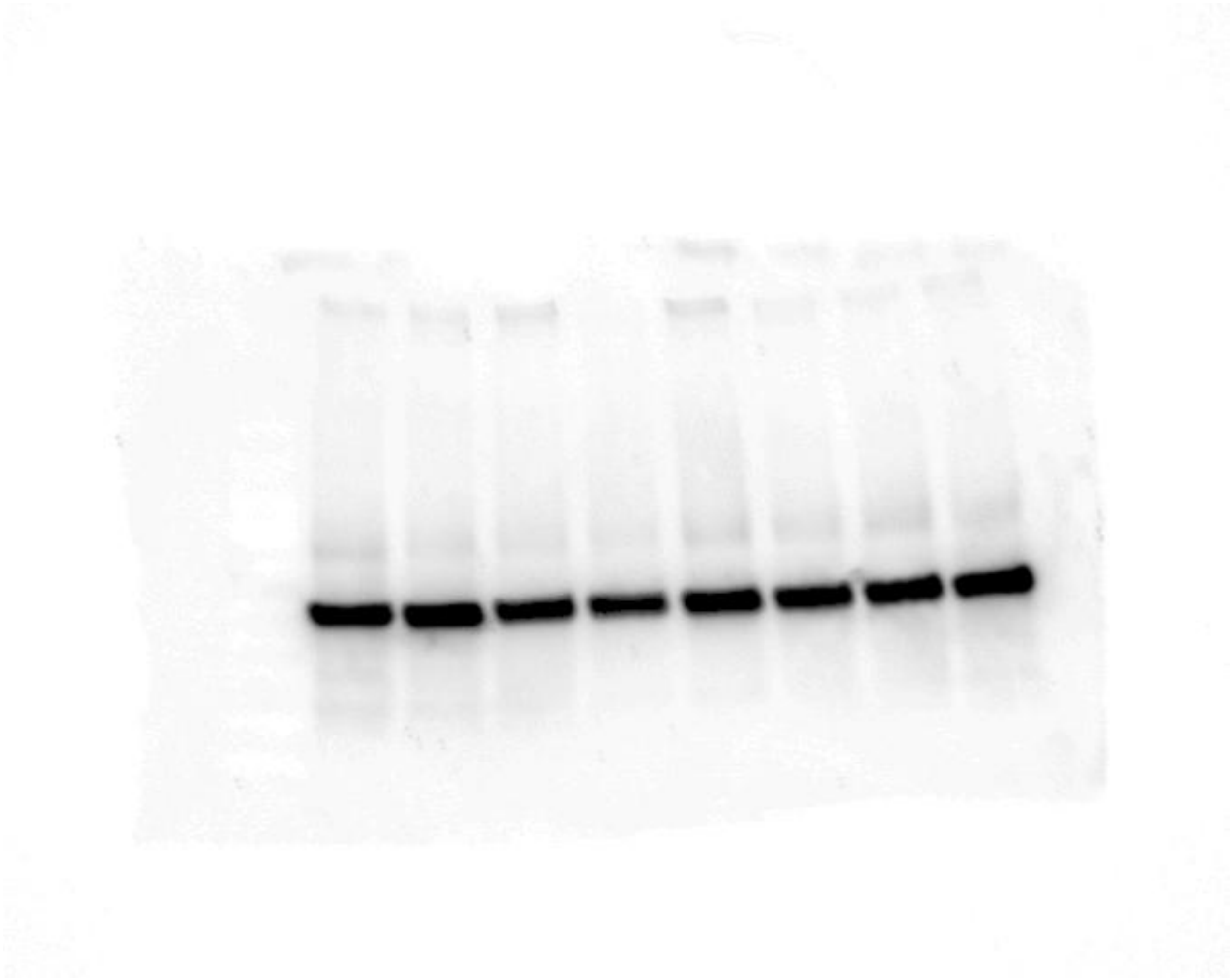

MW Marker:

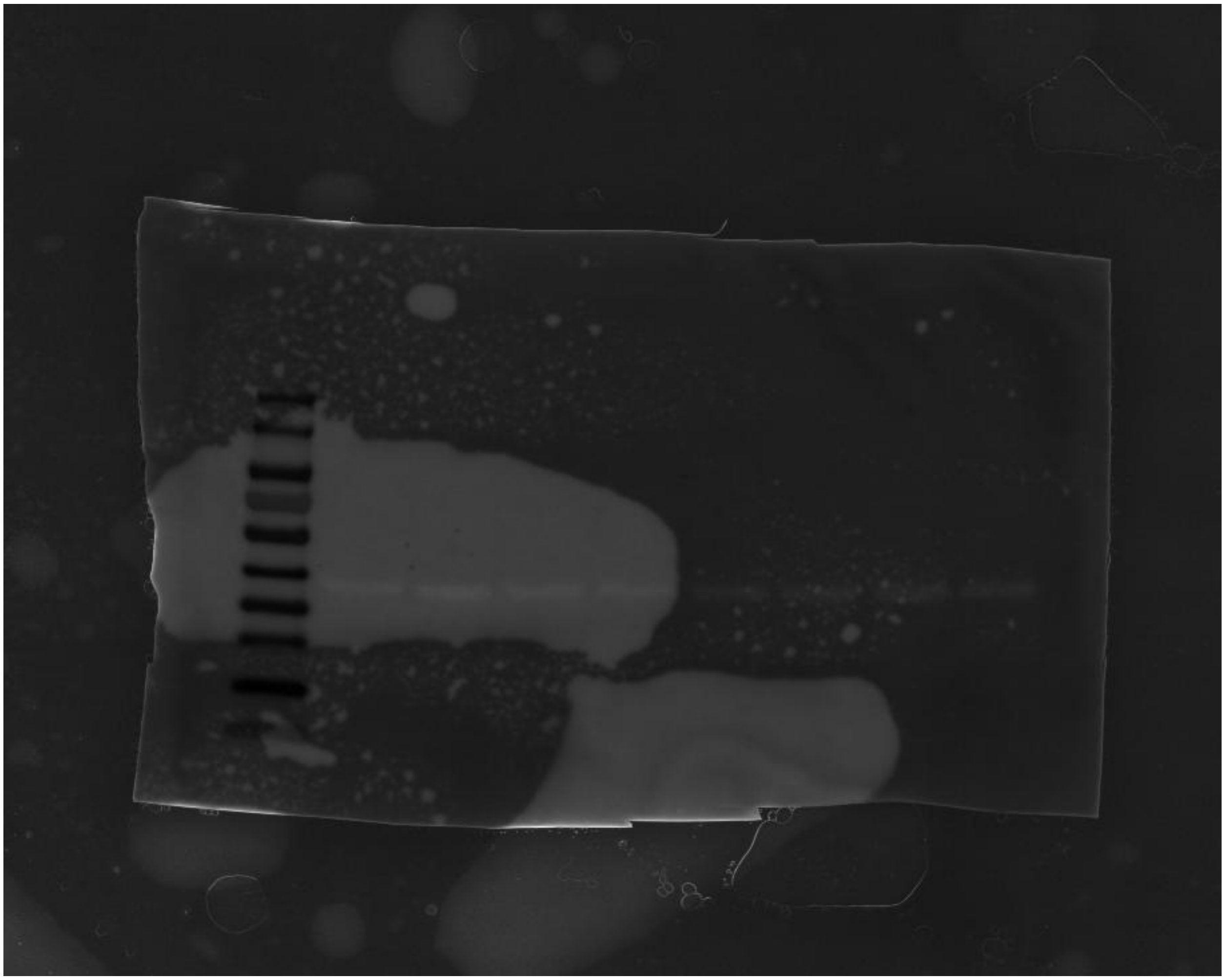

Supplement: Supplementary file 6 — Source Data for Expanded View [file EMMM-11-e9423-s010.zip › emmm201809423-sup-0010-SDataEV/source_data_for_fig_EV7B.pdf]
