# Supplementary figures and images for: Repeat‐associated non‐AUG translation in C9orf72‐ALS/FTD is driven by neuronal excitation and stress
Source: EMBO Mol Med. 2019 Jan 7;11(2):e9423. doi: 10.15252/emmm.201809423 (PMC6365928; doi:10.15252/emmm.201809423)

# Pre-processed Images

GA-188

GP-188

GR-188

PA-188

PG-188

PR-188

Dendra2

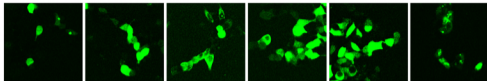

NES-mIFP

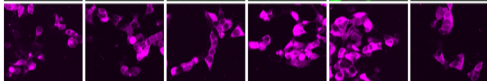

# Post-processed Images

Dendra2

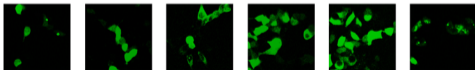

Merged

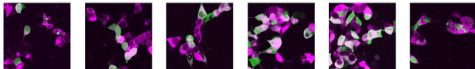

Supplement: Supplementary file 6 — Source Data for Expanded View [file EMMM-11-e9423-s010.zip › emmm201809423-sup-0010-SDataEV/source_data_for_fig_1C_and_EV1C.pdf]

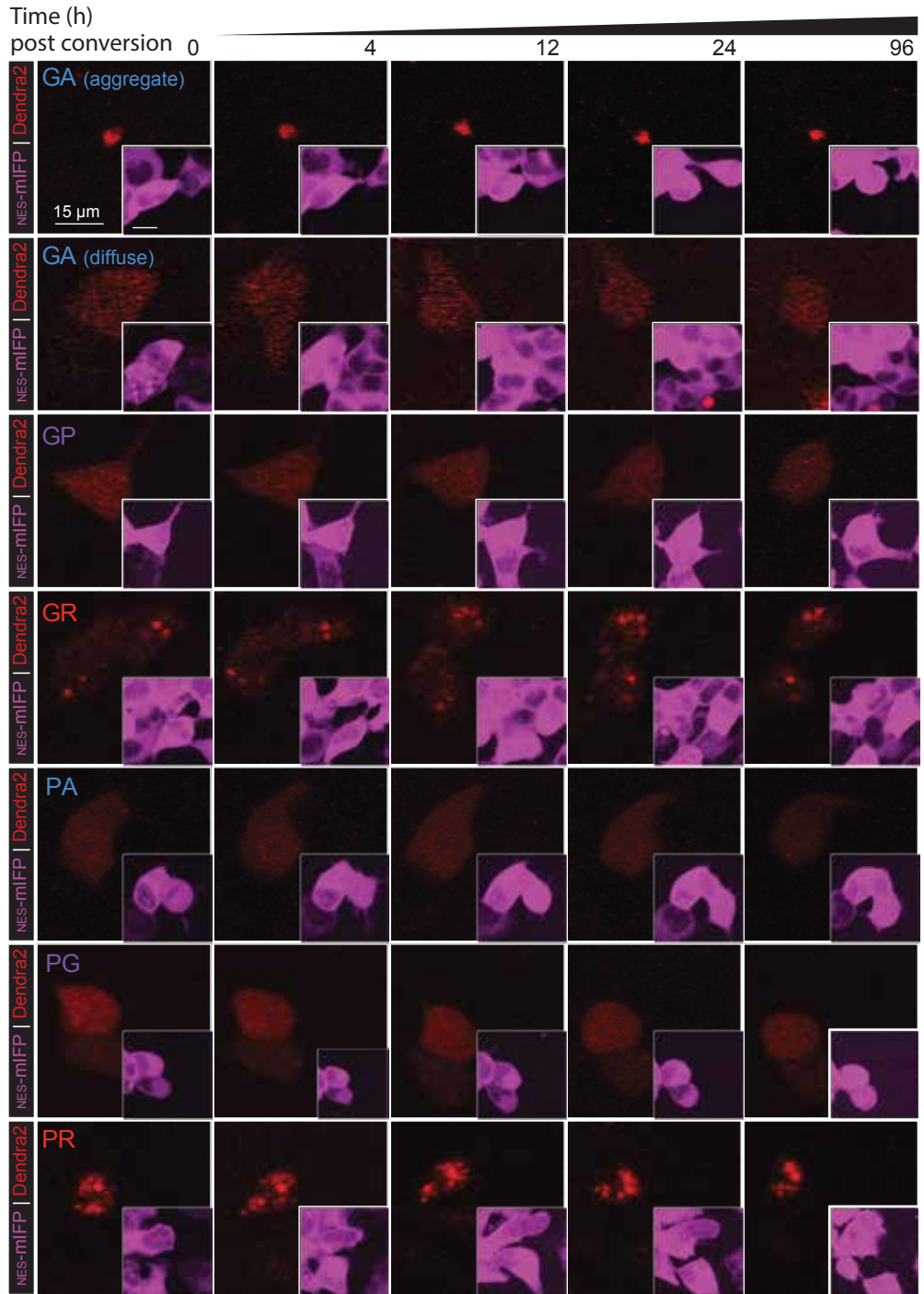

Supplement: Supplementary file 6 — Source Data for Expanded View [file EMMM-11-e9423-s010.zip › emmm201809423-sup-0010-SDataEV/source_file_for_EV3.pdf]

EIF2A

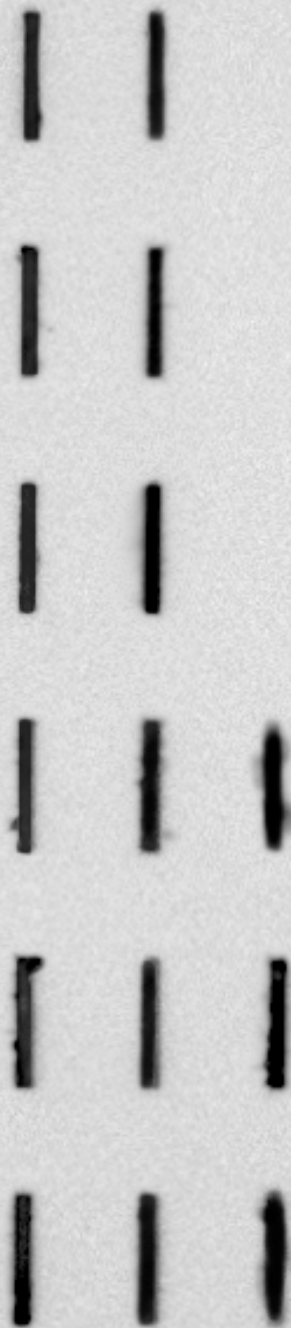

p-eif2a

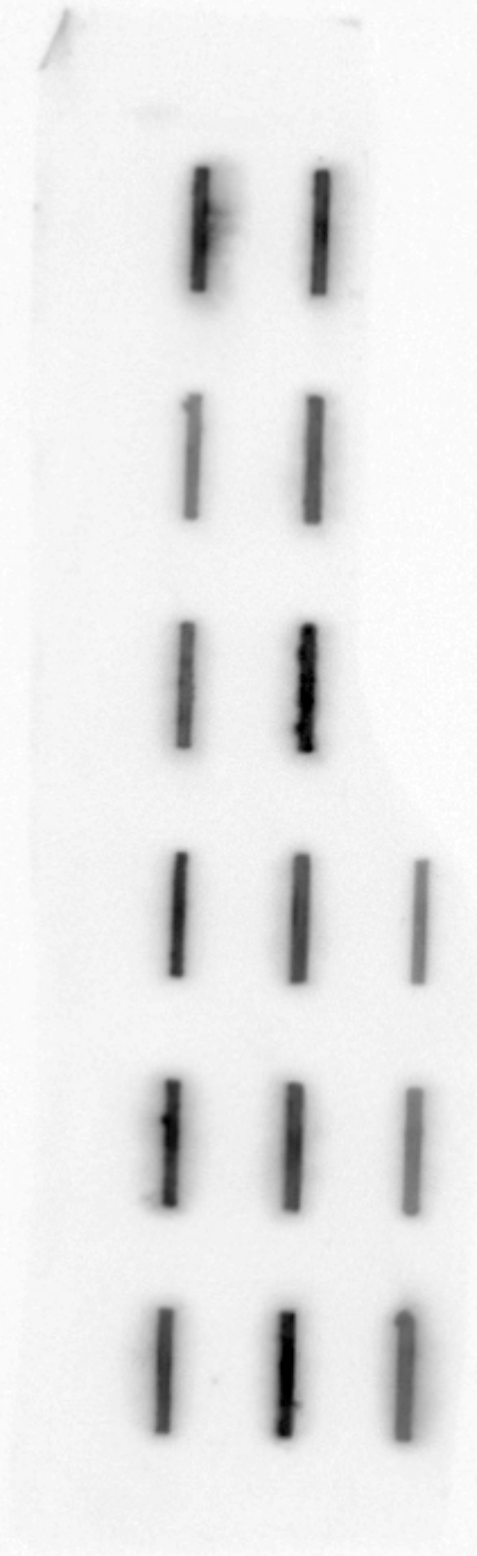

PERK

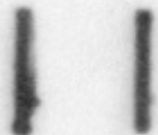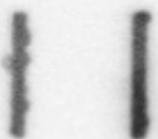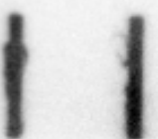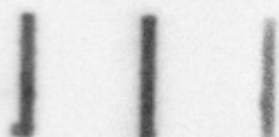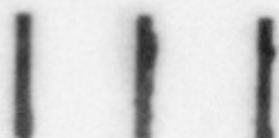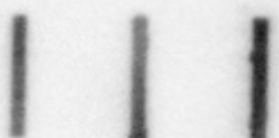

ATF4

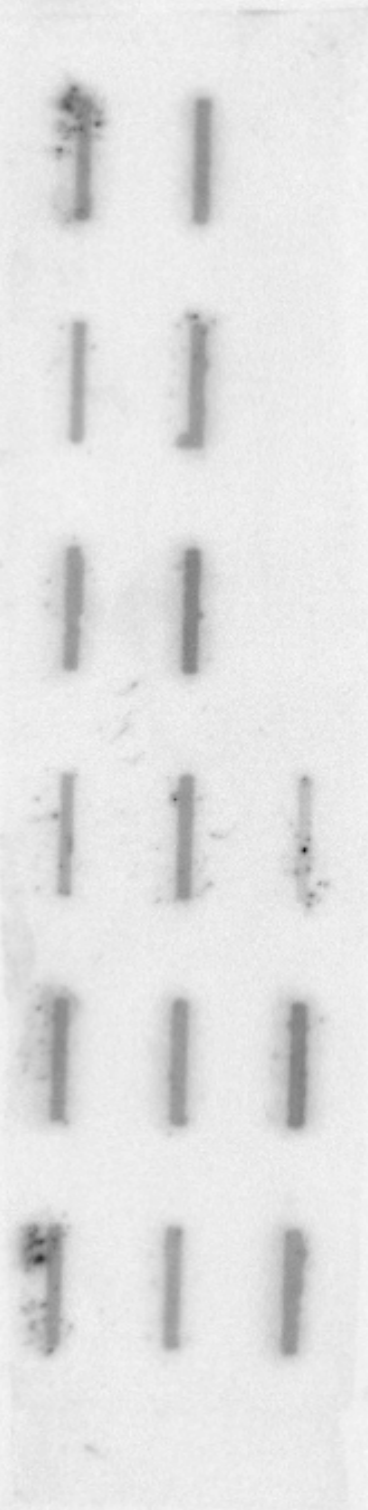

GAPDH

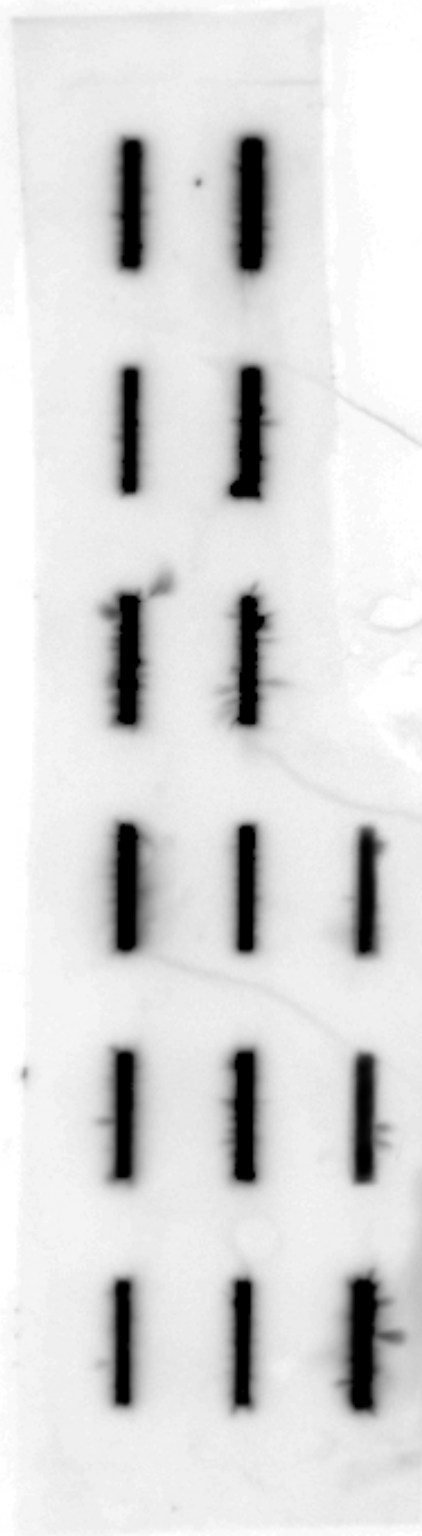

Supplement: Supplementary file 10 — Source Data for Figure 3 [file EMMM-11-e9423-s008.pdf]
