# Supplementary material for: Repeat‐associated non‐AUG translation in C9orf72‐ALS/FTD is driven by neuronal excitation and stress
Source: EMBO Mol Med. 2019 Jan 7;11(2):e9423. doi: 10.15252/emmm.201809423 (PMC6365928; doi:10.15252/emmm.201809423)
Supplement: Supplementary file 8 — Source Data for Figure 1 [file EMMM-11-e9423-s006.pdf]

Figure 1B and EV1B – Representative Filter Trap Assays

Anti-HA: Figure 1B and EV1B

Order of sample – GA, GP, GR, PA, PG, PR

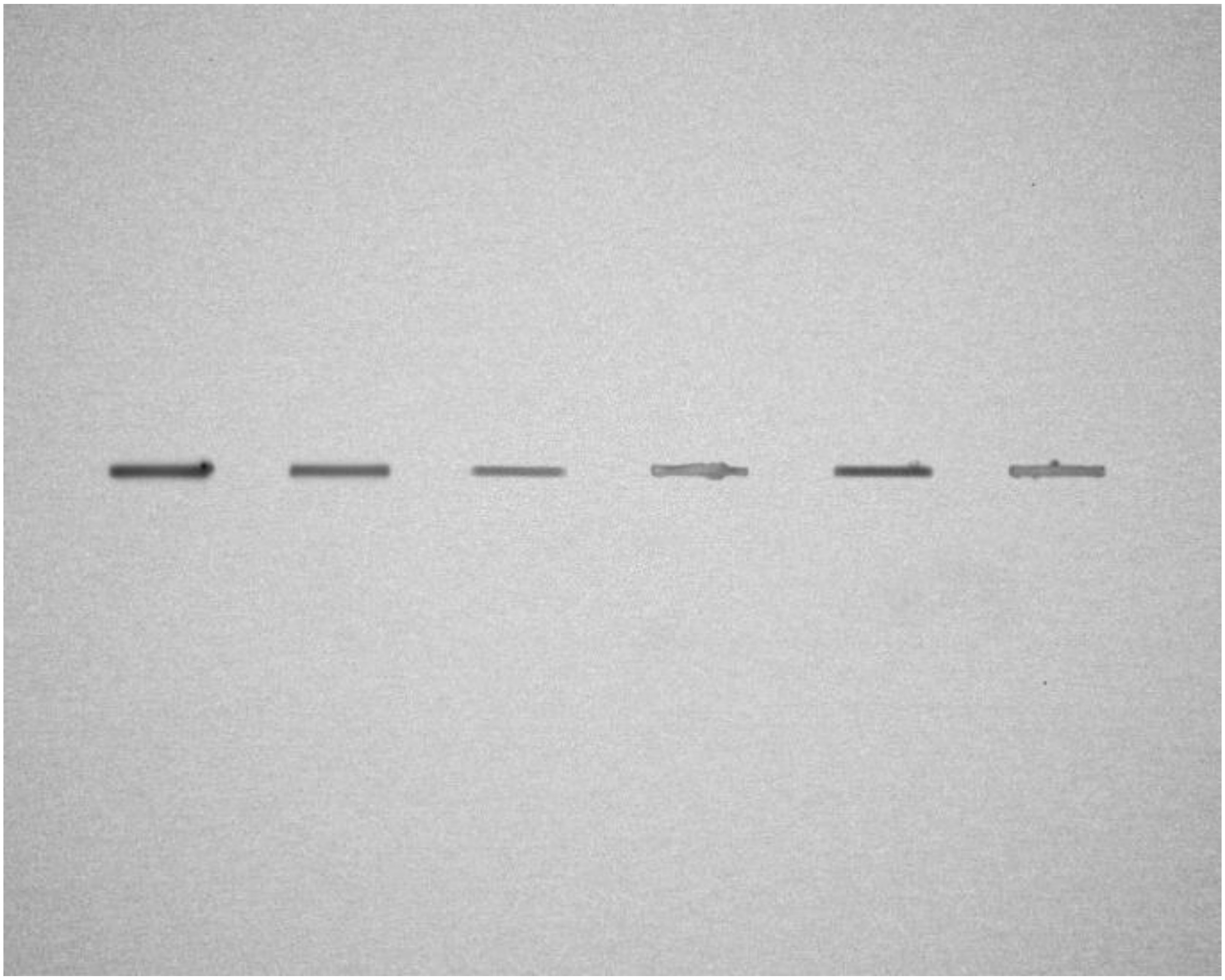

Anti-GAPDH: Figure 1B and EV1B

Same order as above

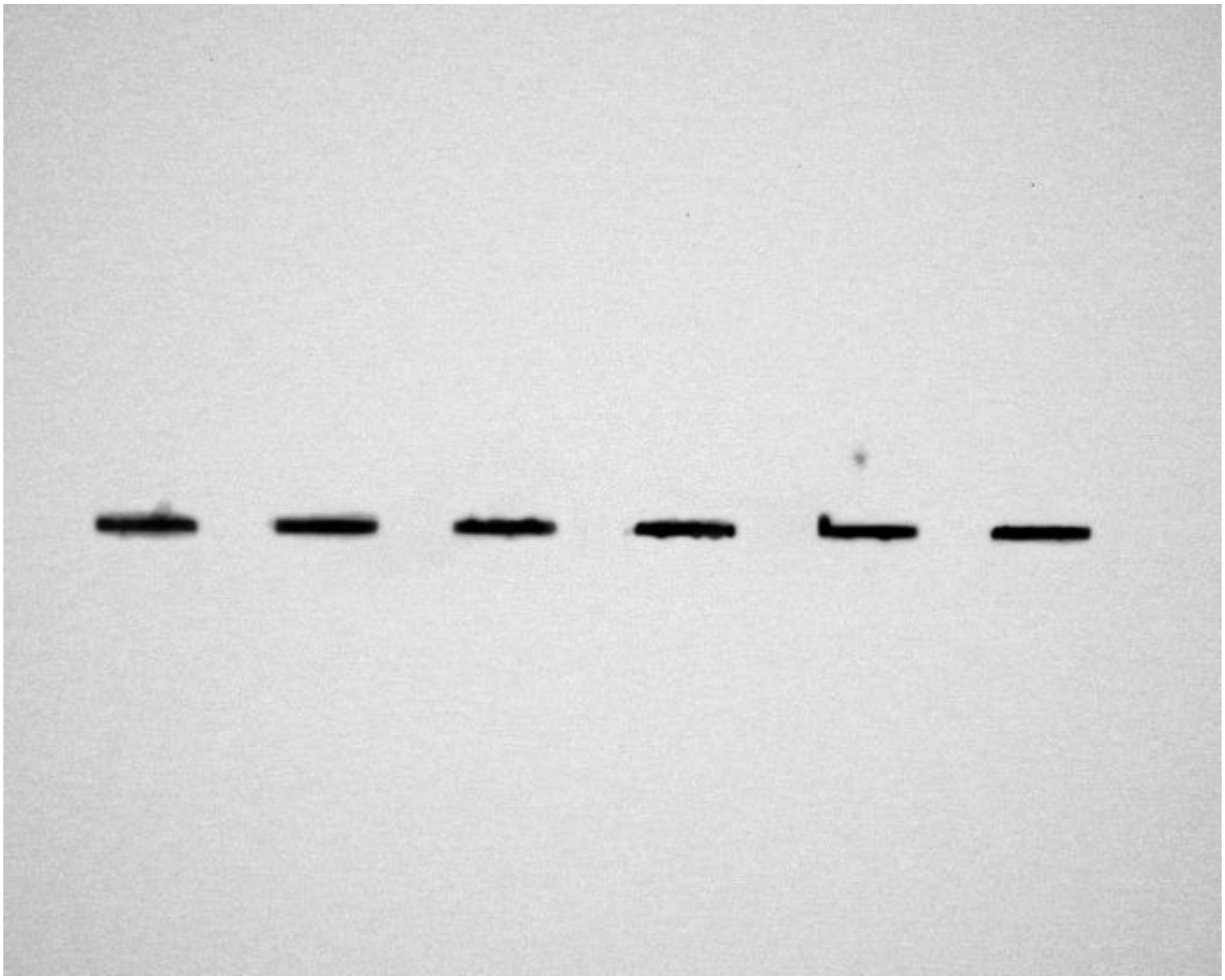

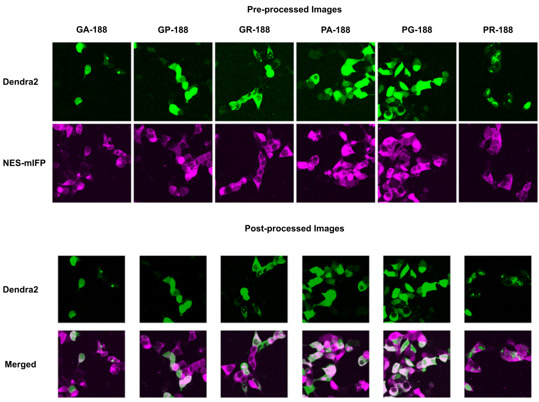
Pre-processed Images
GA-188
GP-188
GR-188
PA-188
PG-188
PR-188
Dendra2
NES-mIFP
Post-processed Images
Dendra2
Merged
