# Supplementary material for: Repeat‐associated non‐AUG translation in C9orf72‐ALS/FTD is driven by neuronal excitation and stress
Source: EMBO Mol Med. 2019 Jan 7;11(2):e9423. doi: 10.15252/emmm.201809423 (PMC6365928; doi:10.15252/emmm.201809423)

Figure 2B Representative blots used in figures that have correct order of conditions

FIg 2 - anti-GA Filter Trap Assay

Line 1 - TG through MS275

Line 2 - Etop through Glut

Line 3 - Positive Ctrl, Ctrl, Negative Ctrl

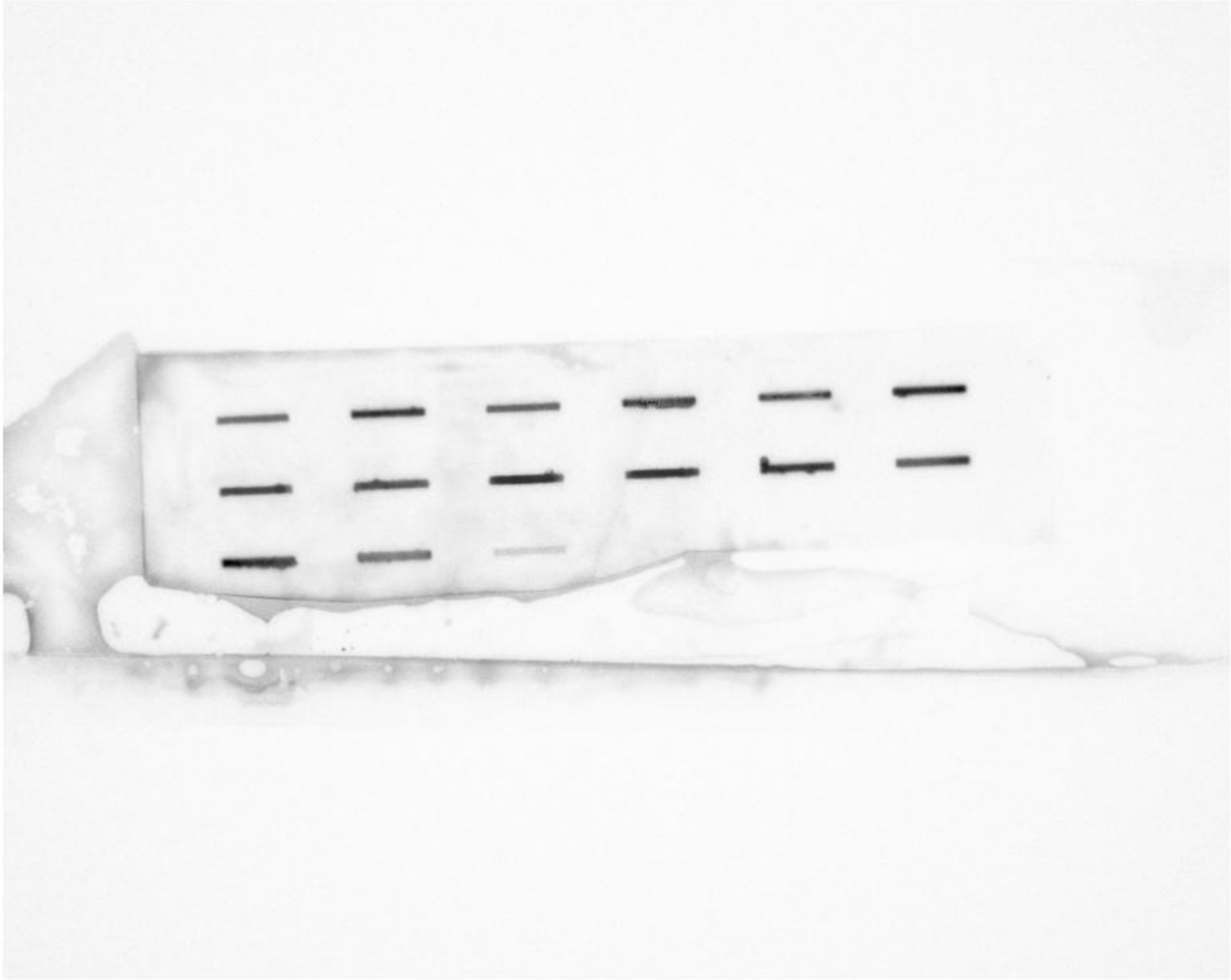

Fig 2 - anti-GP Filter Trap Assay: same order as above

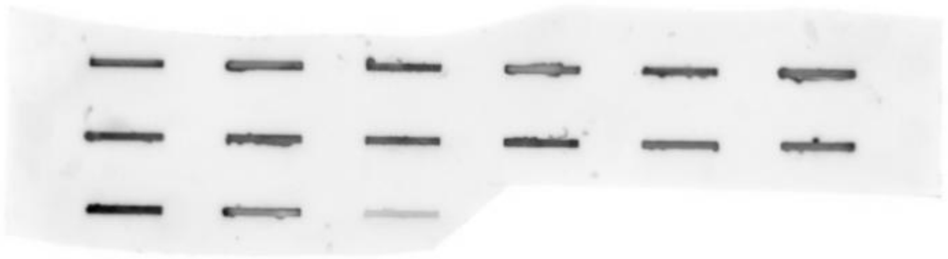

Fig 2 - Anti-GR Filter Trap Assay: same order as above

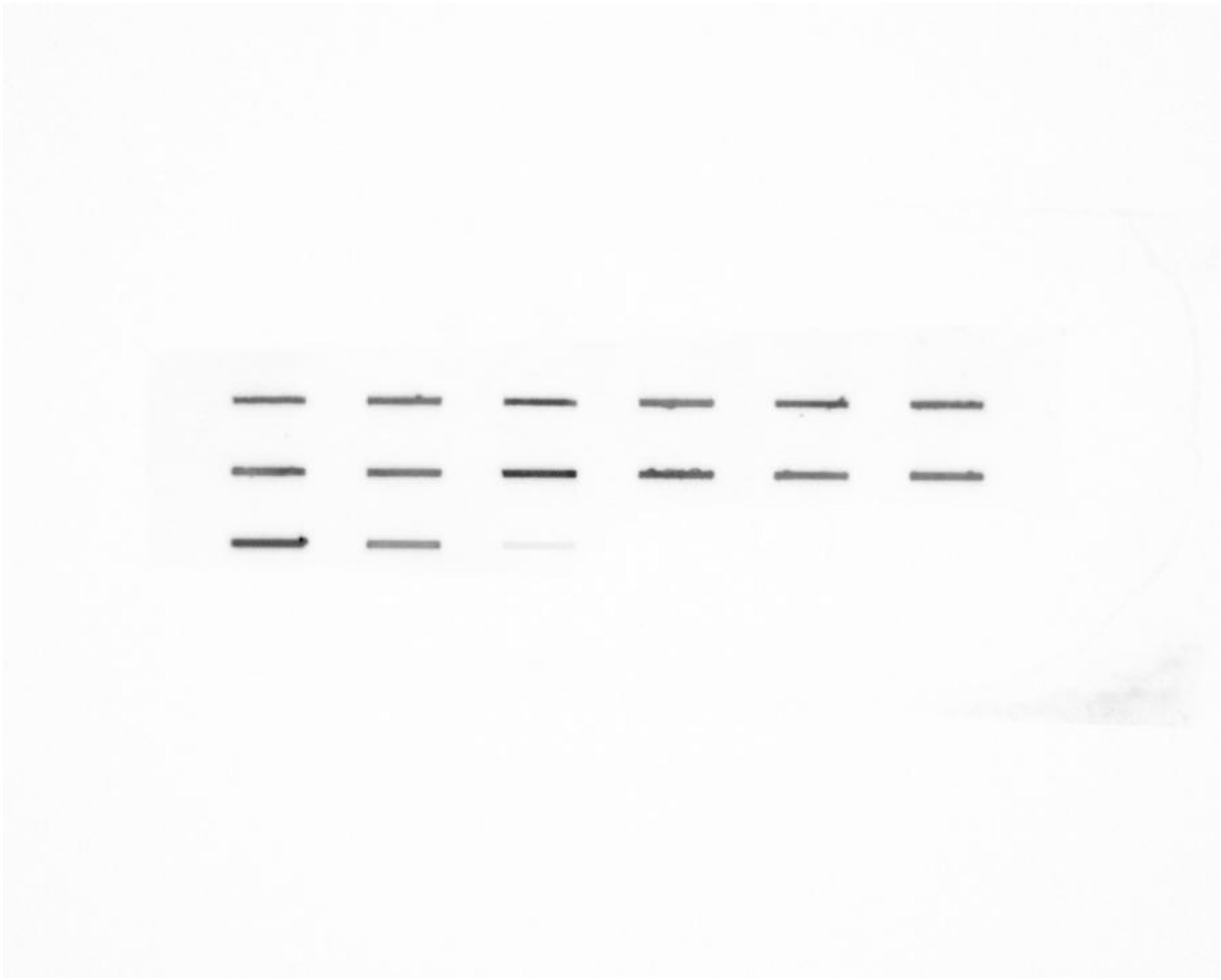

Fig 2 - Anti GAPDH Filter Trap Assay

Line 1 - Not used to represent data, antigen was dispersed from poor pressure

Line 2 - TG through MS275

Line 3 - Etop through Glut

Line 4 - Ctrl

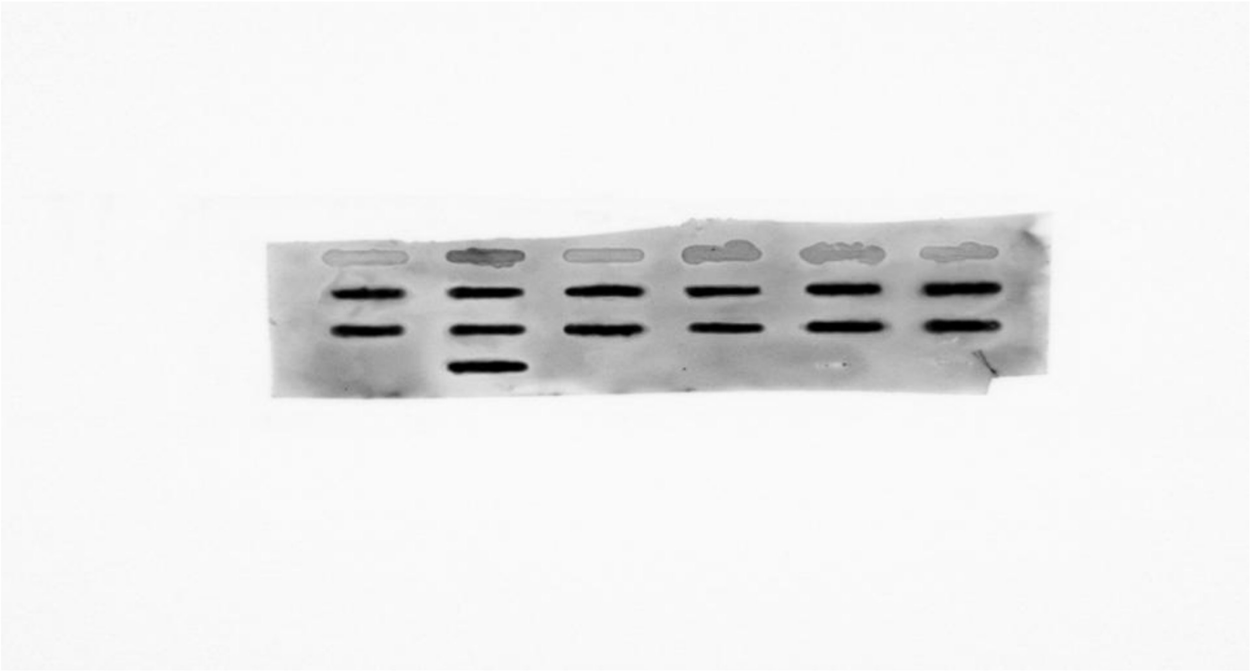

Supplement: Supplementary file 9 — Source Data for Figure 2 [file EMMM-11-e9423-s007.pdf]
