# Supplementary material for: Repeat‐associated non‐AUG translation in C9orf72‐ALS/FTD is driven by neuronal excitation and stress
Source: EMBO Mol Med. 2019 Jan 7;11(2):e9423. doi: 10.15252/emmm.201809423 (PMC6365928; doi:10.15252/emmm.201809423)

Figure 5A and EV5A - Representative blots used in figures that have correct order of conditions FIg 5 - anti-P-eif2α Filter Trap Assay

Line 1 - TG through MS275

Line 2 - Etop through Glut

Line 3 - EV5 HEK, CTRL/ EV5 NSC34, EV5 Neuron

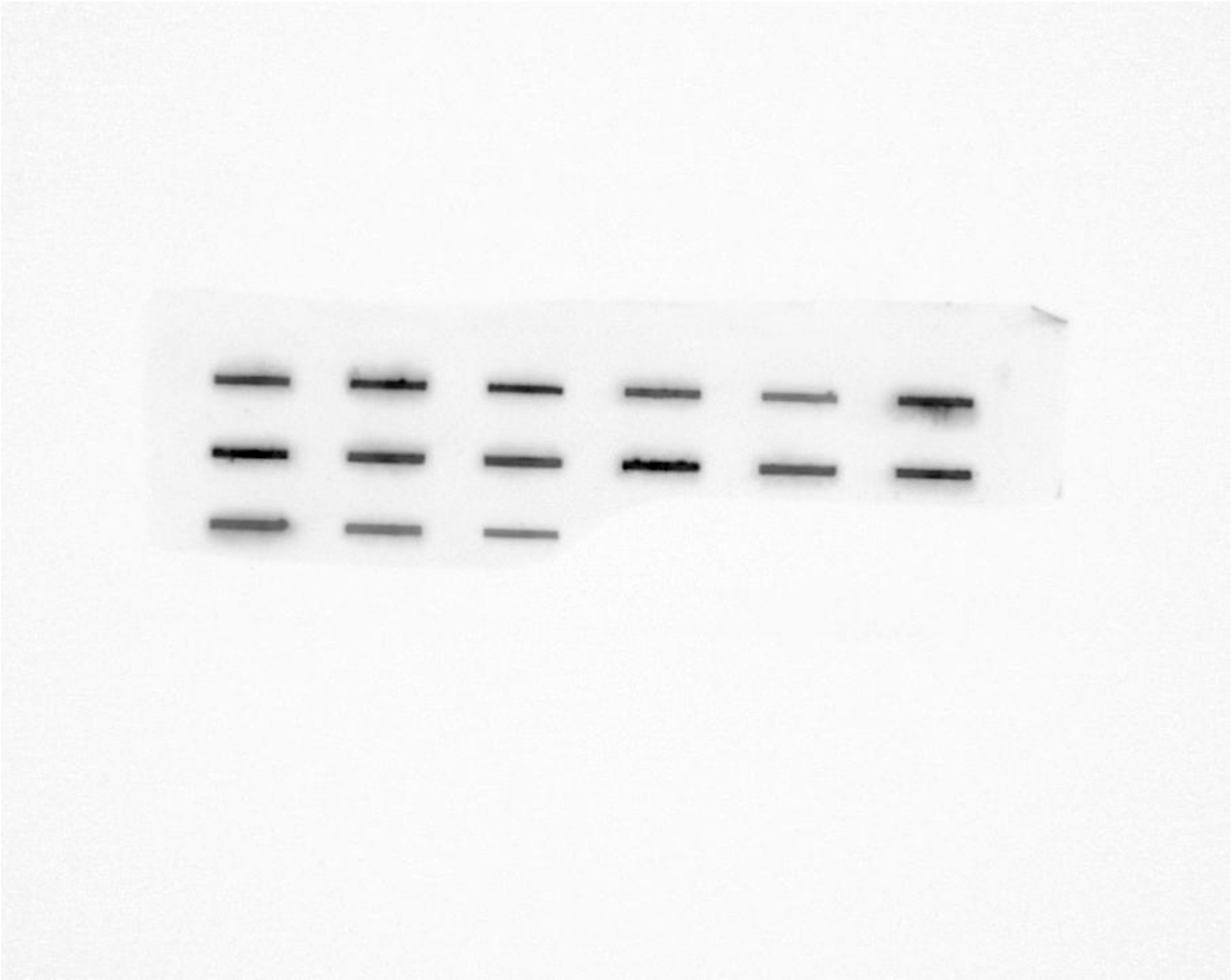

FIg 5 - anti-eif2α Filter Trap Assay

Line 1 - TG through MS275

Line 2 - Etop through Glut

Line 3 - EV5 HEK, CTRL/ EV5 NSC34, EV5 Neuron

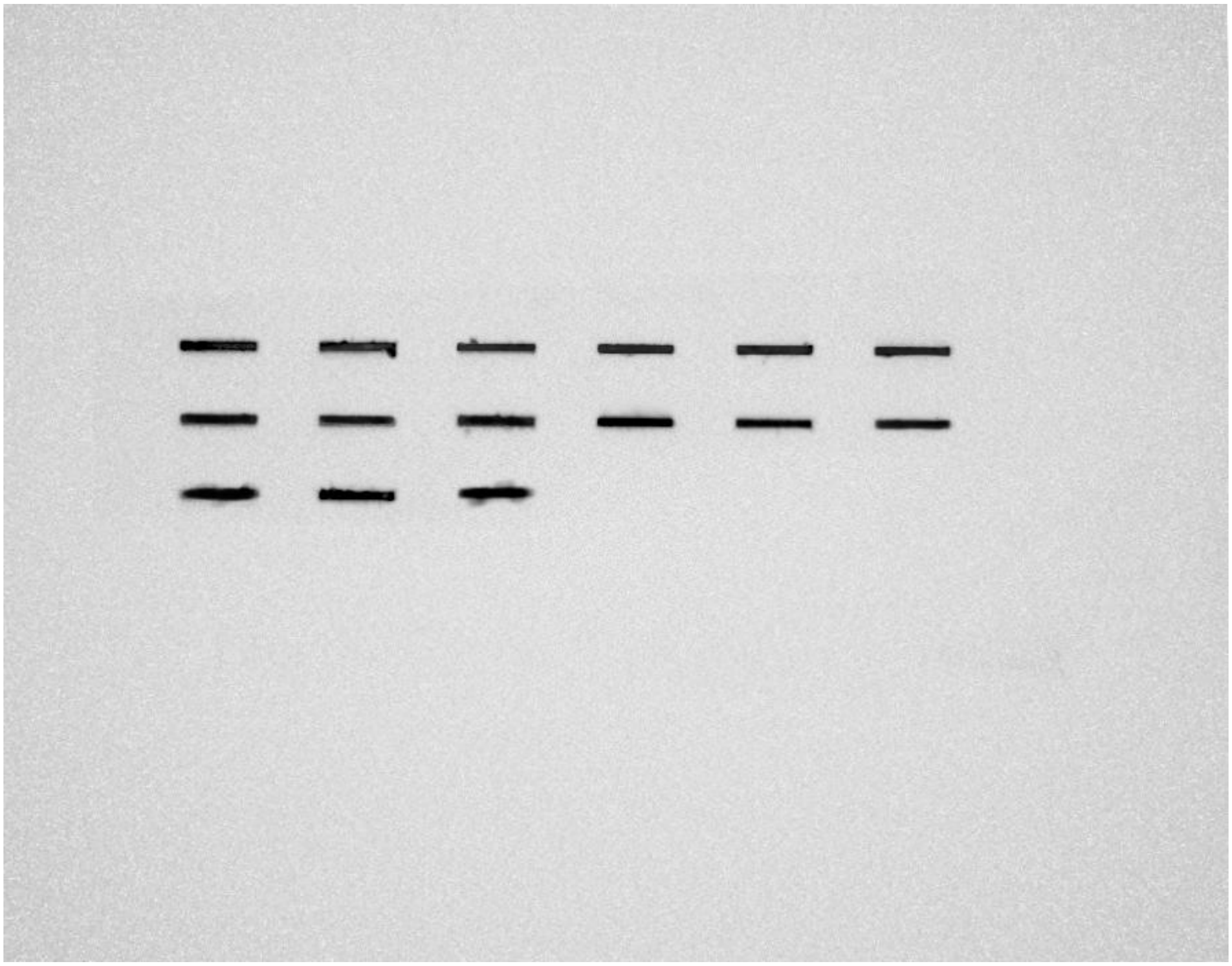

FIg 5 - anti-P-PERK Filter Trap Assay

Line 1 - TG through MS275

Line 2 - Etop through Glut

Line 3 - EV5 Neuron, EV5 HEK, CTRL/ EV5 NSC34

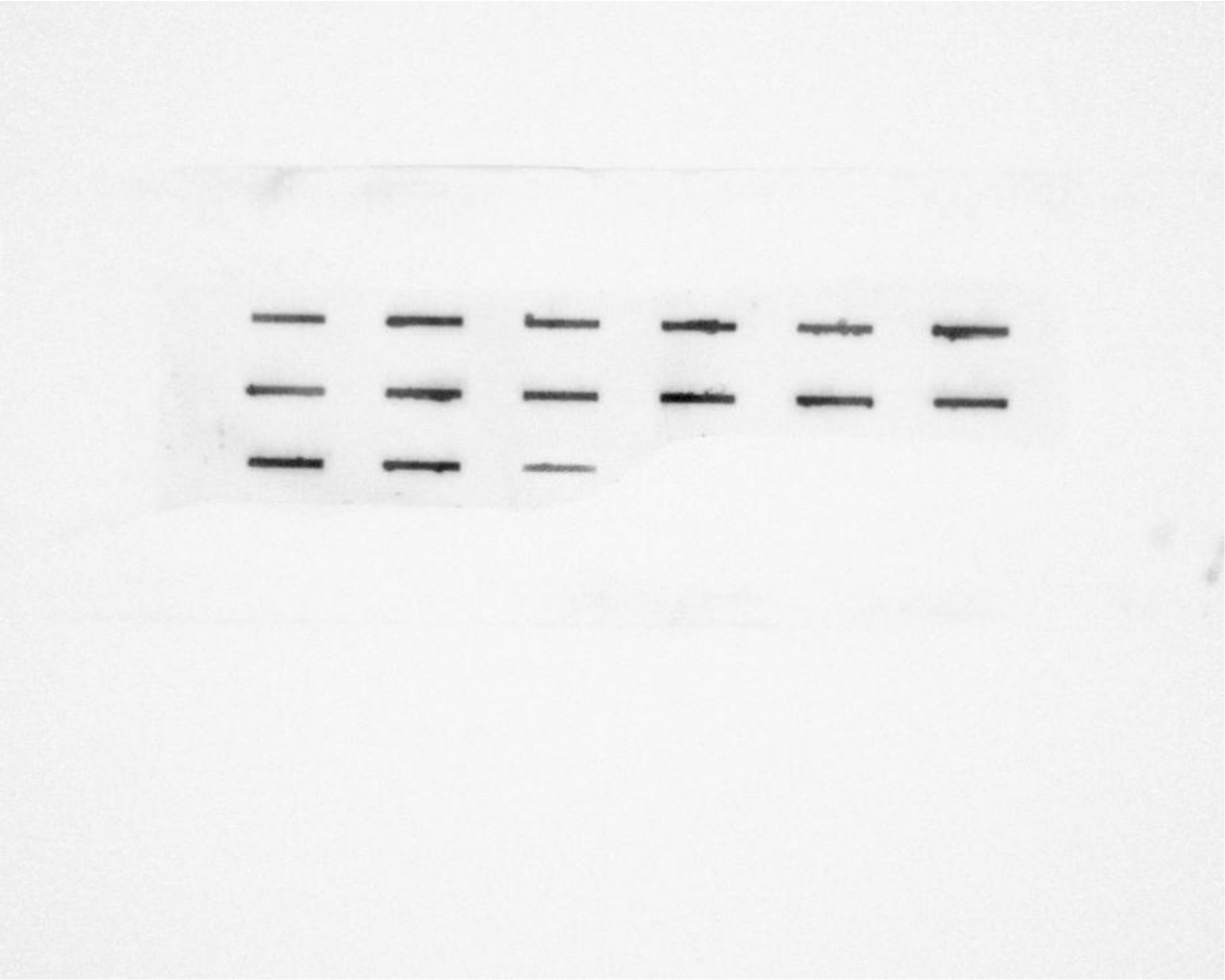

FIg 5 - anti-ATF4 Filter Trap Assay

Line 1 - TG through MS275

Line 2 - Etop through Glut

Line 3 - CTRL/ EV5 NSC34, EV5 HEK, EV5 Neuron

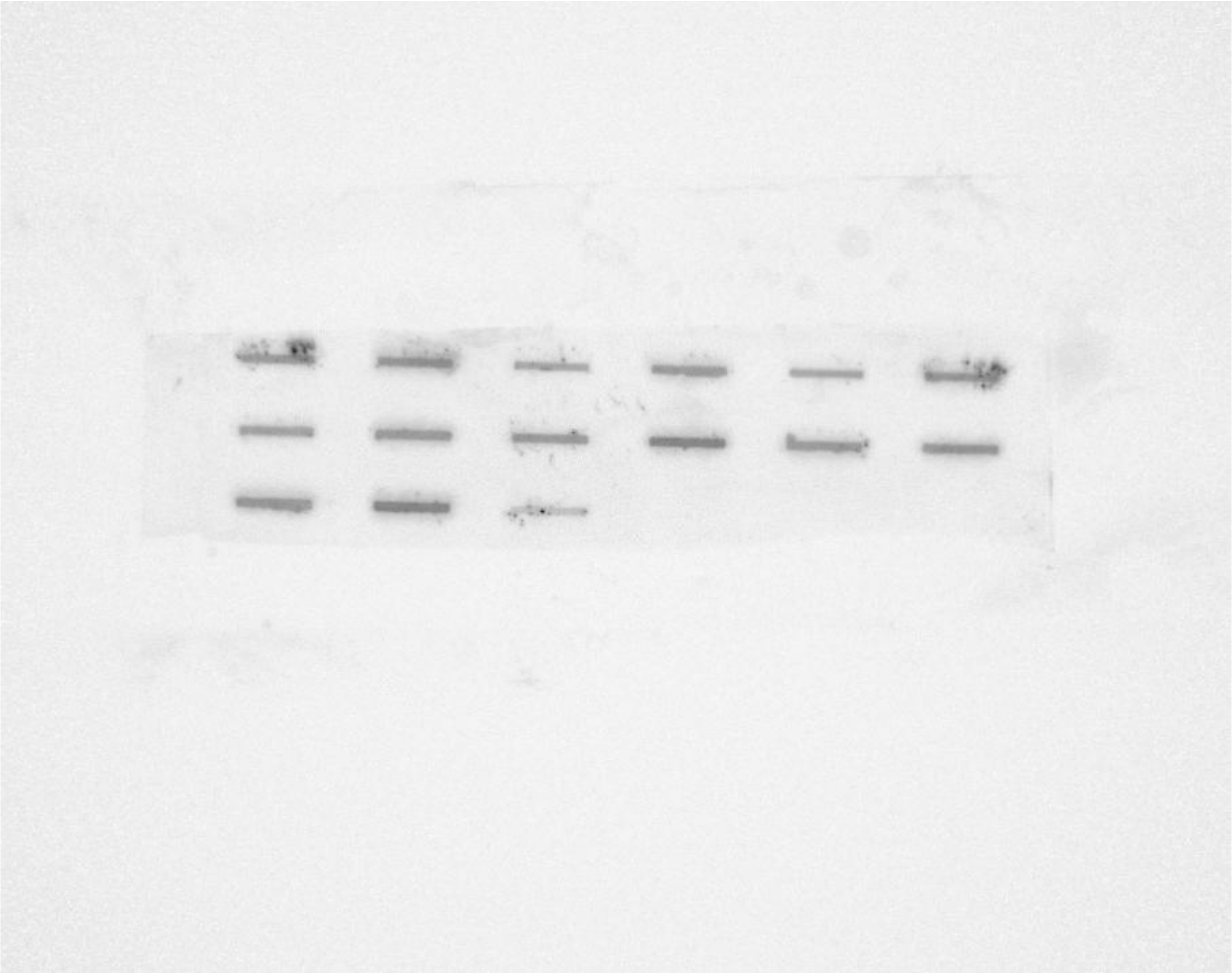

FIg 5 - anti-GAPDH Filter Trap Assay

Line 1 - TG through MS275

Line 2 - Etop through Glut

Line 3 - CTRL/ EV5 NSC34, EV5 HEK, EV5 Neuron

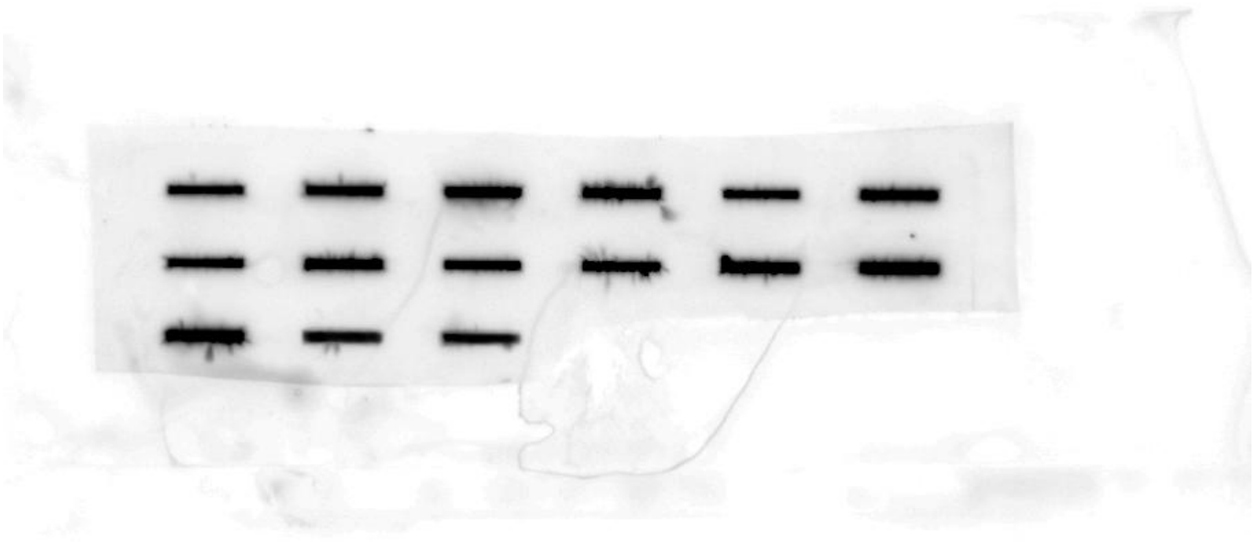

Supplement: Supplementary file 11 — Source Data for Figure 5 [file EMMM-11-e9423-s009.pdf]
